# Insecticidal and Ovicidal Activity of *Cymbopogon citratus* Essential Oil and Its Nanoemulsion Against Hemipteran Crop Pests with Mortality, Antennal Malformations, and Volatile Alterations

**DOI:** 10.3390/insects16121254

**Published:** 2025-12-10

**Authors:** Raul V. C. Apolinário, Jefferson D. Cruz, Walter S. M. F. Neto, Janaína M. C. Soares, Maria A. Mpalantinos, Suzete Araujo Oliveira Gomes, Denise Feder, José L. P. Ferreira, Geraldo J. N. Vasconcelos, Jefferson R. A. Silva, Ana Claudia F. Amaral

**Affiliations:** 1 Laboratório de Plantas Medicinais e Derivados, Farmanguinhos, Fundação Oswaldo Cruz, Rio de Janeiro 21041-250, RJ, Brazil; apolinario_raul@yahoo.com (R.V.C.A.); jefferson_dacruz@hotmail.com (J.D.C.); maria.mpalantinos@fiocruz.br (M.A.M.); 2 Laboratório de Cromatografia, Departamento de Química, Instituto de Ciências Exatas, Universidade Federal do Amazonas, Manaus 69077-000, AM, Brazil; wssottoo@gmail.com (W.S.M.F.N.); janaina25soares02@gmail.com (J.M.C.S.); 3 Departamento de Biologia Geral—GBG, Instituto de Biologia, Universidade Federal Fluminense, Niterói 24210-201, RJ, Brazil; suzetearaujo@id.uff.br (S.A.O.G.); mdfeder@id.uff.br (D.F.); 4 Departamento de Farmácia e Administração Farmacêutica, Faculdade de Farmácia, Universidade Federal Fluminense, Niterói 24241-000, RJ, Brazil; josepint06@yahoo.com.br; 5 Instituto de Ciências Exatas e Tecnologia, Universidade Federal do Amazonas, Itacoatiara 69103-128, AM, Brazil; gjnvasconcelos@ufam.edu.br

**Keywords:** *Euschistus heros*, *Dysdercus peruvianus*, lemongrass oil, scanning electron microscopy, olfactometer, HS-SPME

## Abstract

Hemipteran insects such as *Euschistus heros* and *Dysdercus peruvianus* are important pests of soybean and cotton, respectively, making them relevant targets in pest management programs. This work aims to evaluate the insecticidal and chemical activity of *Cymbopogon citratus* essential oil (CC-EO) and its nanoemulsion against *E. heros* and *D. peruvianus*. A mixture of citral stereoisomers (59.5%) was identified as the major constituent of CC-EO The topical application of nanoemulsion resulted in 53.33% and 33.33% mortality in *E. heros* and *D. peruvianus*, respectively. Contact treatment caused 83.33% mortality in *E. heros* and 86.70% mortality in *D. peruvianus*, also inducing antennal deformities observed by scanning electron microscopy and reflected in altered behavioral responses in a Y-tube olfactometer assay. Ovicidal effects were observed in contact treatment for both species, affecting nymph emergence from eggs treated with nanoemulsion. Treatments with pure EO resulted in 100% nymph mortality and complete inhibition of egg hatching in both insects. Headspace solid-phase microextraction (HS-SPME) analysis revealed changes in the volatile profile of treated insects, showing the presence of semiochemicals related to defense and alarm, such as terpenes and alcohols. The multiple lethal and sublethal effects demonstrated by CC-EO highlight its potential as a candidate for integrated pest management programs, offering an eco-friendly alternative to conventional chemical control methods.

## Simple Summary

Insects of *Dysdercus peruvianus* and *Euschistus heros* are considered pests in the respective plantations of cotton and soybean, resulting in crop damage and productivity losses. The use of essential oils (EOs) as potential insecticides represents a sustainable pest-control method and an eco-friendly approach for reducing environmental impacts caused by the use of conventional pesticides. Nanoemulsion systems can improve the interaction of EOs with water, enhancing their solubility and biological activity. The *Cymbopogon citratus* EO and its nanoemulsion were tested against *E. heros* and *D. peruvianus*, showing mortality effects via topical and contact treatments, as well as ovicidal effects. Other results consisted of modifications of insect morphology by deformities in antennae, alterations in the behavior of insects, and changes in the volatile profile of semiochemicals. The insecticidal and chemical activities demonstrated by *C. citratus* EO and nanoemulsion against *D. peruvianus* and *E. heros* highlight their potential as candidates for programs of hemipteran pest control.

## 1. Introduction

Agriculture plays an important role in feeding the population and providing raw materials to industry, including food, fibers, and fuel [1,2,3]. The production of these materials directly impacts the economy, with estimates that improvements in crop productivity account for one-third of the global economy, corresponding to 4.3 trillion dollars [4,5]. Soybean and cotton cultivation represent a significant addition to global crop production, generating 348.86 million tons of soybeans and 41.6 million tons of cotton seeds; furthermore, only in Brazil, production amounts to 120.7 million tons and 3.4 million tons for soy and cotton, respectively [6].

However, this significant economic and productive potential faces constant threats posed by phytophagous organisms in agroecosystems, particularly in strategic crops such as soybean and cotton. In this context, insects considered crop pests reach high population levels, causing damage to plantations, reflected in crop losses and affecting the cost of control measures [7,8]. Estimates show that pests are responsible for 20–40% of global annual productivity loss [9]. In Brazil, insects may cause production losses of 4.31 million tons for soybeans and 155.5 thousand tons for cotton, precipitating a total economic loss of 14.7 million dollars [10].

The suborder Heteroptera, represented by an estimated diversity of 88 recognized families and 45,000 species, includes numerous insects of agricultural importance [11]. Among them, sap-sucking bug pests threaten soybean and cotton by damaging plant tissues and transmitting pathogens through feeding sites [12,13]. *Euschistus heros* (Fabricius, 1798), belonging to the Pentatomidae family, is considered a polyphagous insect that feeds on plant parts from different families and is characterized as one of the main agricultural pests in South America, reducing soybean [*Glycine max* (L.) Merill (Fabaceae)] crops [14,15]. *Dysdercus peruvianus* (Guérin-Méneville, 1831), belonging to the Pyrrhocoridae family, is known for damaging cotton [*Gossypium hirsutum*, (Malvaceae)] seeds and fibers, causing stains due to deposition of waste that can also spread viruses, fungi, and bacteria [16].

Given the significance of these phytophagous insects on an agricultural scale, an analysis of effective control methods based on Integrated Pest Management (IPM) initiatives is crucial. Although IPM goals tend to reduce pesticide use, these products still represent the main chemical control method for crop protection, with large global use in agriculture estimated at 3.70 million tons of active ingredients [17]. Products such as acephate (organophosphate), imidacloprid, and acetamiprid (neonicotinoids) are commonly used to control *E. heros* and *Dysdercus* species [18,19,20]. These insecticides have also reported to be toxic to non-target organisms and mammals [21,22,23].

Considering the drawbacks of conventional pesticides, such as toxicity, environmental persistence, and insect resistance, there is increasing demand for safer and more sustainable alternatives. In this context, essential oils (EOs), complex mixtures of volatile plant-derived compounds such as terpenes and phenylpropanoids, have gained prominence as eco-friendly biopesticides. They exhibit low toxicity to non-target organisms, minimal environmental persistence, and reduced potential for resistance development in pest species [24,25,26,27].

Despite these advantages, EOs are limited by their high volatility, low aqueous solubility, and susceptibility to degradation under environmental conditions [28]. Advances in green biotechnology and nanotechnology have led to the development of nanoemulsion-based formulations, which enhance EO stability, solubility, and bioavailability. These systems enable efficient interaction between aqueous and oily phases without relying on toxic organic solvents, thereby reducing environmental impact and minimizing bioaccumulation risks [29,30,31].

In this context, the Poaceae family is recognized for its considerable biodiversity, comprising approximately 789 genera and 11,783 accepted species, and within this family, the genus *Cymbopogon* comprises nearly 59 distinct species [32]. *Cymbopogon citratus* (D.C.) Stapf, popularly known as lemongrass, is cultivated worldwide for its therapeutic and medicinal properties, and its products have shown several biological activities, including antiviral, antifungal, antitumor, insecticidal, and antioxidant [33,34,35].

Although the biological potential of lemongrass essential oil is well documented, studies on the biological activity of its nanoemulsions against agriculturally important hemipteran species remain scarce. The present study evaluates the biotechnological potential of *Cymbopogon citratus* essential oil (CC-EO) and its nanoemulsion through their insecticidal activity against *Euschistus heros* and *Dysdercus peruvianus*, as well as their potential influence on semiochemical expression in treated insects. The findings highlight a promising application in IPM programs for soybean and cotton crops, offering an eco-friendly strategy for controlling key agricultural pests.

## 2. Materials and Methods

### 2.1. Reagents and Standards

The surfactants Tween 20, Tween 80, and Span 80 were purchased from Sigma-Aldrich (St. Louis, MO, USA). Distilled and Milli-Q ultrapure water were used for general procedures. Triflumuron insecticide Hades SC (50.52% triflumuron) was obtained from Dominus (Jandaia do Sul, PR, Brazil). Solvents (Tedia, Rio de Janeiro, Brazil) were used for analytical and HPLC-grade chromatography.

### 2.2. Plant Material and Essential Oil Extraction

Specimens of *C. citratus* were cultivated in experimental plots at the Laboratory of Biomass Production and Plant Raw Material of Farmanguinhos (Fiocruz). For taxonomic identification, samples were sent to the Rio de Janeiro Botanical Garden (JBJR), where the corresponding voucher was authenticated and deposited in the herbarium under voucher number RB377575.

Fresh leaves of *C. citratus* (300 g) were collected, weighed, and fragmented. The plant material was immersed in 3 L of distilled water and hydrodistilled for 2 h using a Clevenger-type apparatus (Marconi, Piracicaba, Brazil). The essential oil (EO) was recovered following aqueous-phase drainage, centrifuged at 2000 rpm for 4 min to remove residual water, transferred to amber glass ampoules, and stored at low temperature until analyses and assays. The EO yield was calculated as a function of the fresh weight of plant material used.

### 2.3. Nanoemulsion Preparation and Characterization

Nanoemulsion formulations were prepared using the low-energy phase inversion method [36]. Four different formulations of CC-EO were developed, each with distinct hydrophilic–lipophilic balance (HLB) values: 15 (formulations A and B), 14, and 13 (Table 1) All formulations were composed of 5% (*w*/*w*) essential oil, 5% (*w*/*w*) surfactant mixture, and 90% (*w*/*w*) Mili-Q water, for a final volume of 2 mL per formulation. The aqueous phase was gradually added to the oil phase under continuous stirring. In formulation A (HLB 15), the surfactant system consisted of 86 mg of Tween 20 and 14 mg of Span 80. Formulation B (also HLB 15) used 100 mg of Tween 80 alone. The HLB 14 formulation included 90.65 mg of Tween 80 and 9.35 mg of Span 80, while the HLB 13 formulation used 81.3 mg of Tween 80 and 18.7 mg of Span 80. Each formulation contained 100 mg of EO and 1800 mg of Mili-Q water.

Preliminary analyses were conducted 24 h post-preparation to assess physical stability, including the presence of phase separation and creaming. HLB 15 formulations showed phase separation, while the HLB 13 formulation exhibited a creamy appearance. The HLB 14 formulation presented a slightly bluish-white, translucent appearance, characterized by the Tyndall effect, and was selected for further characterization and bioassays. A blank nanoemulsion was also prepared using the HLB 14 surfactant system, but without EO.

For characterization, 10 µL of each nanoemulsion (EO-loaded and blank) was diluted in 3.9 mL of Mili-Q water and analyzed via dynamic light scattering (DLS) using a Litesizer DLS 500 (Anton Paar, Gras, Austria). Particle size, polydispersity index (PDI), and zeta potential were monitored at 25 °C, over 90 days.

### 2.4. Insect Colonies and Biological Assays

Colonies of *E. heros* and *D. peruvianus* were established at the Laboratory of Insect Biology (UFF), reared at 25 ± 1 °C and 60% relative humidity as described by Mourão and Panizzi, and Gonzalez et al. [37,38]. Genetic access was registered in SISGEN under authorizations A394AA4 (*E. heros*) and A0E95C4 (*D. peruvianus*).

Fourth-instar nymphs were randomly selected for topical and contact exposure to evaluate molting to fifth instar and subsequent adult metamorphosis over 20 days after treatment (DAT). Mortality and sub-lethal effects were recorded. Each group included 10 insects, with 9 replications (*n* = 90).

For topical assays, 1 μL of CC-EO or its nanoemulsion (100 mg EO/2 mL) was applied to the dorsal cuticle of insects [39]. Nanoemulsion treatment corresponded to 50 µg EO per insect. Control groups included blank nanoemulsion and untreated insects, and triflumuron (1 μL, diluted in 1 mL distilled water) was used as a positive control [40].

In contact assays, 50 μL of CC-EO or nanoemulsion (containing 2.5 mg EO; 39.3 µg/cm^2^) was applied in ten 5 μL aliquots to filter paper in 9 cm Petri dishes (63.6 cm^2^). After 3 min of drying, nymphs were added, and dishes were sealed with parafilm. Controls included blank nanoemulsion, untreated filter paper, and 50 μL of triflumuron (1 mg/mL) as a positive control.

For ovicidal assays, adult insects were transferred to separate containers after completing metamorphosis to allow mating and oviposition. Eggs collected after 10 days were used in contact treatments. Groups of 20 eggs (*n* = 180 per treatment) were exposed to treated filter paper with 50 μL of CC-EO, nanoemulsion, blank nanoemulsion, triflumuron, or left untreated. Hatching was monitored daily up to 10 DAT.

### 2.5. Scanning Electron Microscopy (SEM)

Fifth-instar nymphs of *Dysdercus peruvianus* (10 DAT) and adults of *Euschistus heros* (15 DAT) exhibiting antennal malformations following exposure to CC-EO nanoemulsion were selected for SEM analysis. The untreated group served as a control, while the blank nanoemulsion group was excluded because it showed no morphological alterations. Insects were fixed in 2.5% glutaraldehyde (*v*/*v*) in 0.1 M sodium cacodylate buffer (pH 7.2) and rinsed three times (10 min each) in the same buffer. Dehydration was performed using an ethanol gradient (7.5%, 15%, 30%, 50%, 70%, and 90%), followed by three steps in 100% ethanol, each for 10 min. Samples were then dried using critical point CO_2_ (Autosamdri R-815, Tousimis, Rockville, MD, USA), sputter-coated with gold (Denton Vacuum Desk IV, Denton Vacuum LLC, Moorestown, NJ, USA), and examined under a JEOL JSM-6390LV scanning electron microscope (JEOL Ltd., Akishima, Japan). Antennal segments and trichoid sensilla (Ts) were evaluated for structural alterations according to the criteria described in the literature [41].

### 2.6. Olfactometer Assays

Behavioral assays were performed using a custom-built Y-tube olfactometer (30 cm stem, 20 cm arms, and 4 cm diameter) [42]. The setup was positioned on a white surface to minimize external visual interference, and a lamp was directed at the Y-junction to enhance internal illumination. A Maxxi Power Pro-9000 air pump (Maxxi Power, São Paulo, Brazil) generated airflow through Teflon tubing connected to glass flasks containing activated charcoal and distilled water for filtration. The purified air was regulated to 0.5 L/min using flow meters and directed to odor source chambers (glass tubes, 8 cm long, and 4 cm in diameter) at the ends of each arm.

A total of 90 unsexed fourth-instar *D. peruvianus* nymphs were subjected to contact treatment with CC-EO nanoemulsion. At 10 DAT, surviving individuals that molted to the fifth instar and exhibited antennal deformities were selected (*n* = 30) for behavioral assessment. For comparison, 30 untreated fifth-instar nymphs with normal antennae were also tested. Due to insufficient numbers of malformed individuals, *E. heros* was not included in the olfactometer assays.

Filter paper squares (20 × 20 mm) placed inside each odor chamber received 10 µL of the test solutions. Two binary-choice tests were conducted: (i) CC-EO vs. liquid paraffin, and (ii) CC-EO nanoemulsion vs. blank nanoemulsion. Individual insects were released at the base of the Y-tube and observed for a maximum of 2 min. A choice was recorded when the insect fully traversed the stem and entered either arm. Non-responding insects (those that remained in the stem beyond 2 min without entering an arm) were noted as such. Each insect represented a single replicate. To avoid positional bias, odor sources were alternated between arms every five replicates.

All assays were conducted under controlled conditions (25 ± 1 °C, 60% relative humidity). The glass components of the olfactometer were cleaned with 70% ethanol and dried between tests involving treated and untreated insects.

### 2.7. Gas Chromatography Coupled to Mass Spectrometry: Characterization of Essential Oil and Volatile Compounds

The chemical profile of CC-EO was determined using GC–MS (Agilent 6890N coupled to 5973N quadrupole MS, Santa Clara, CA, USA; DB-5MS column, 30 m × 0.25 mm × 0.25 µm). Injections (1.0 µL, splitless mode) were made at 250 °C with helium as the carrier gas (0.5 mL/min). The oven was programmed from 40 °C to 300 °C at 4 °C/min. Detection parameters included an ion source at 230 °C, electron ionization at 70 eV, and a scan range of m/z 40–700. Compound identification was based on retention indices and mass spectra comparison with published data and the Wiley library.

For insect volatile analysis, *E. heros* and *D. peruvianus* nymphs from the CC-EO nanoemulsion group (with antennal malformations) and untreated controls were selected at key timepoints (10 DAT for *D. peruvianus* during peak molting; 15 days for *E. heros* at peak metamorphosis). Specimens (*n* = 5 per group/species) were frozen and subjected to headspace solid-phase microextraction (HS-SPME). A 65 µm PDMS/DVB fiber was used, preconditioned by immersion in a methanol/water (4:1) solution for 30 min, followed by heating to 250 °C for 10 min in the GC injector.

For extraction, insects were sealed in cleaned 20 mL vials and incubated at 40 °C in a 100 mL water bath for 10 min. A CAR/PDMS fiber was exposed to the headspace for 1 h and immediately desorbed into the GC–MS for analysis.

Volatile compounds were analyzed using a Shimadzu GC-2010 system (Shimadzu Corporation, Kyoto, Japan) coupled to a QP2010 Plus MS (DB-5MS column, 30 m × 0.25 mm × 0.25 µm). The oven program started at 40 °C (1 min), ramped at 5 °C/min to 120 °C (10 min hold), then 7 °C/min to 240 °C (5 min hold). Helium was used as the carrier gas (50 mL/min); the injector, interface, and detector were maintained at 250 °C, 240 °C, and 280 °C, respectively. Components were identified by comparing retention indices and fragmentation patterns to the Adams library and instrument spectral databases [43].

### 2.8. Statistical Analysis

GraphPad Prism version 9.3.0 was used to generate graphs and perform statistical analysis. Analysis of variance (ANOVA) and Tukey’s test were used for analysis of insecticidal results and sub-lethal effects; data were expressed in *F*(DFn, DFd) values and *p* values. The chi-square test was performed to analyze response rate in the olfactometer, with results expressed as χ^2^ (Df) and *p* value. Differences between the untreated control and treated groups were considered significant when *p* < 0.05.

## 3. Results

### 3.1. Essential Oil Characterization and Chemical Constituents

Gas chromatography analysis identified four major components in CC-EO. The most abundant was citral, a mixture of the isomers geranial and neral (59.52%), followed by cis-linalool oxide (21.75%) and trans-linalool oxide (18.73%).

### 3.2. Nanoemulsion Characterization

Figure S1 shows the initial parameters of HLB 14 formulations. EO nanoemulsion had a particle size of 242 nm and a zeta potential of −22.1 mV (Figure S1A,C), while blank nanoemulsion had a particle size of 195.11 nm and a zeta potential of −9.6 mV in zeta potential (Figure S1B,D). Analysis of the nanoemulsions over a 90-day period revealed variations in particle size, PDI, and zeta potential for both the CC-EO (HLB 14)-prepared formulation and the blank formulation (HLB 14). The initial PDI values were 0.272 for the CC-EO nanoemulsion and 0.277 for the blank. On day 1, the particle size of the CC-EO nanoemulsion was 242.0 nm, increasing progressively to 358.7 nm by day 90. The PDI fluctuated over time, starting at 0.159, peaking at 0.275, and the zeta potential ranged from −22.1 mV to −19.8 mV throughout the period. In the blank nanoemulsion, the initial particle size was 195.11 nm, increasing to 349.3 nm by day 90. The PDI started at 0.277, reached a low of 0.213, and then slightly increased to 0.239. Zeta potential values showed a marked increase in negativity, from −9.6 mV at the start to −30.7 mV by the end of the 90-day period. These results indicate a trend of particle size enlargement and variability in colloidal stability parameters for both nanoemulsion types over time.

### 3.3. Insecticidal Assays

**Figure 1:**
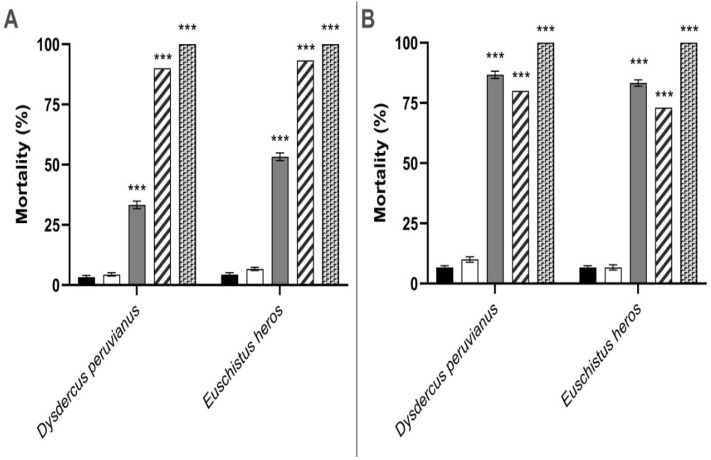
Mean percentage of *Euschistus heros* and *Dysdercus peruvianus* insects’ mortality after 20 days of treatment with *Cymbopogon citratus* essential oil (CC-EO) and nanoemulsion: (**A**)—topical treatment; (**B**)—contact treatment. 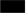 = Untreated control; 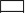 = Blank nanoemulsion; 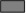 = CC-EO nanoemulsion; 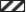
= Positive control (triflumuron); 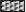 = Pure CC-EO. *** Statistically significant difference compared with the untreated control (*p* < 0.0001; Tukey’s test, *n* = 10 insects in 9 replicates per treatment).

In the topical treatment, ANOVA analysis demonstrated *F*(4,40) = 232.7 for *E. heros* data and *F*(4,40) = 212.7 for *D. peruvianus* data. In Figure 1A, CC-EO displayed 100% mortality (*p* < 0.0001) for both insect species. After 20 DAT, CC-EO nanoemulsion treatment resulted in mortality rates of 53.33 ± 1.58% (*p* < 0.0001) for *E. heros* and 33.33 ± 1.58% (*p* < 0.0001) for *D. peruvianus*. Blank nanoemulsion did not affect insect development, reaching mortality levels of 6.70 ± 0.71% (*p* > 0.05) in *E. heros* and 4.44 ± 0.73% (*p* > 0.05) in *D. peruvianus*. *E. heros* untreated control demonstrated 4.44 ± 0.73% mortality, while *D. peruvianus* presented 3.33 ± 0.71% mortality. Positive controls showed 93.33 ± 0.71% (*p* < 0.0001) and 90.00 ± 1.00% (*p* < 0.0001) mortality in *E. heros* and *D. peruvianus*, respectively.

For contact treatment, ANOVA analysis yielded *F*(4,40) = 180.0 and *F*(3,32) = 146.8 for *D. peruvianus* and *E. heros*, respectively. Figure 1B shows that mortality in untreated controls was 6.70 ± 0.71% for both species. Blank nanoemulsion treatment resulted in mortality rates of 10.0 ± 1.19% (*p* > 0.05) for *D. peruvianus* and 6.70 ± 1.19% (*p* > 0.05) for *E. heros*. The treatment with CC-EO nanoemulsion reached mortality levels of 86.70 ± 1.5% (*p* < 0.0001) and 83.33 ± 1.32% (*p* < 0.0001) in *D. peruvianus* and *E. heros*, respectively. Mortality in the positive control group was 80.00 ± 1.00% (*p* < 0.0001) for *D. peruvianus* and 73.33 ± 1.58% (*p* < 0.0001) for *E. heros*, whereas in the CC-EO-treated group, 100% mortality was observed in both insects.

Table 2 presents the eclosion rates of *E. heros* and *D. peruvianus* eggs subjected to contact treatment with CC-EO and its nanoemulsion. ANOVA data were *F*(4,40) = 90.28 for *E. heros* and *F*(4,40) = 70.61 for *D. peruvianus*.

In the untreated controls, mean eclosion rates at 10 DAT were 83.35% for *E. heros* and 78.35% for *D. peruvianus*. Exposure to the CC-EO nanoemulsion significantly reduced eclosion, with values of 36.10% for *E. heros* and 15.00% for *D. peruvianus*. In contrast, the blank nanoemulsion showed no statistically significant effect compared with controls, with eclosion rates of 75.00% and 70.00% for *E. heros* and *D. peruvianus*, respectively (*p* > 0.05). The positive control (triflumuron) reduced eclosion to 18.35% for *E. heros* and 12.75% for *D. peruvianus*, demonstrating consistent ovicidal activity.

Notably, treatment with pure CC-EO resulted in complete inhibition of egg hatching in both species (*p* < 0.0001). The data indicate a more pronounced reduction in hatching rates in *D. peruvianus* than in *E. heros* across all treatments, particularly with the EO nanoemulsion and positive control.

### 3.4. Insect Deformities

The percentage of deformities in both insect species was calculated based on the total number of insects per treatment group (*n* = 90), and results are presented in Table S1.

Treatments with CC-EO nanoemulsion resulted in deformities in treated insects, totaling 68.8% in *D. peruvianus* and 41.1% in *E. heros*. Contact treatment with EO nanoemulsion resulted in a high proportion of deformities, especially in *D. peruvianus* antennae (64.44%; *p* < 0.0001), followed by *E. heros* antennae deformities (36.67%; *p* < 0.0001). Other developmental abnormalities included insects remaining within the exuvia after molting/metamorphosis, consisting of 4.44% (*p* > 0.05) for *D. peruvianus* fifth-instar nymphs in contact treatment and 4.44% (*p* > 0.05) in *E. heros* adults from topical treatment. Positive control showed that a significant number of insects remained in exuvia on topical treatment, consisting of fifth-instar nymphs and adults of *D. peruvianus* (45.56%, *p* < 0.0001 for nymphs; 3.33%, *p* > 0.05 for adults) and *E. heros* (17.10%, *p* < 0.0001 for nymphs; 24.30%, *p* < 0.0001 for adults). No deformities were observed in insects treated with CC-EO in both treatments with *E. heros* and *D. peruvianus*.

**Figure 2:**
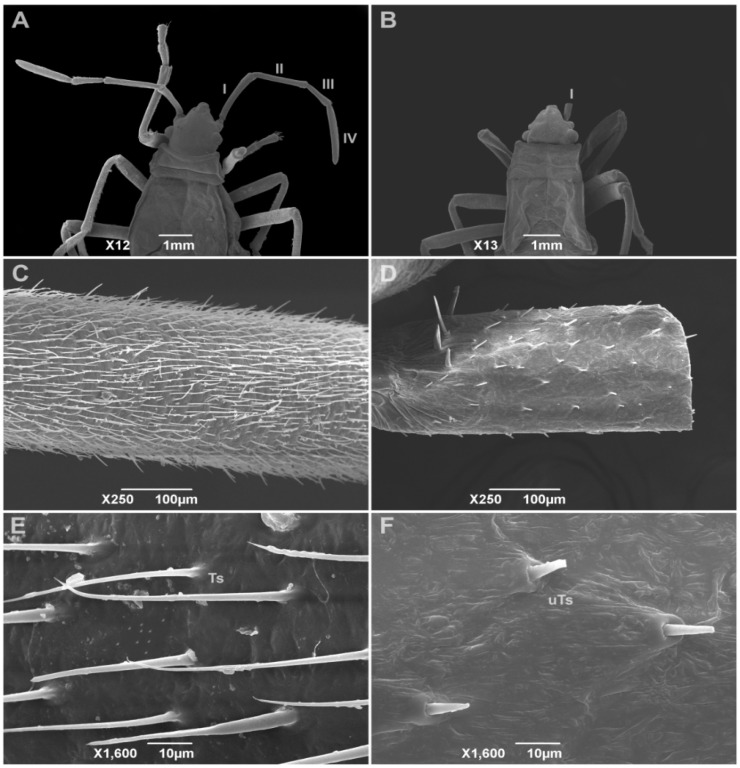
Scanning Electron Micrographs of *Dysdercus peruvianus* insects subjected to contact treatment with *Cymbopogon citratus* essential oil nanoemulsion. (**A**)—fifth-instar insect of untreated control group and its four-segmented antennae (I to IV) (×12); (**B**)—fifth-instar insect from treatment showing only one segment (I) in antenna (×13); (**C**)—segment IV of untreated insect antenna zoomed (×250); (**D**)—segment I of treated insect antenna zoomed (×250); (**E**)—segment IV of untreated insect antenna zoomed (×1600); (**F**)—segment I of treated insect antenna zoomed (×1600). Ts—trichoid sensilla; uTs—undeveloped trichoid sensilla.

**Figure 3:**
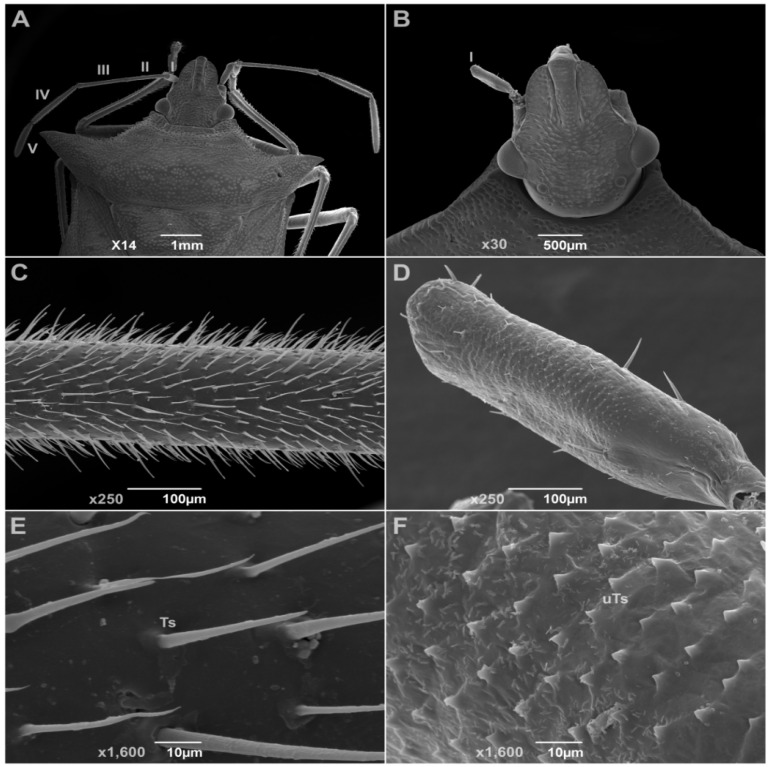
Scanning Electron Micrographs of *Euschistus heros* insects submitted to contact treatment with *Cymbopogon citratus* essential oil nanoemulsion. (**A**)—adult insect of untreated control group and its five-segmented antennae (I to V) (×14); (**B**)—adult insect from treatment showing only one segment (I) in antenna (×30); (**C**)—segment IV of untreated insect antenna zoomed (×250); (**D**)—segment I of treated insect antenna zoomed (×250); (**E**)—segment IV of untreated insect antenna zoomed (×1600); (**F**)—segment I of treated insect antenna zoomed (×1600). Ts—trichoid sensilla; uTs—undeveloped trichoid sensilla.

Figure 2 and Figure 3 represent the SEM observations of *D. peruvianus* and *E. heros* antennae, respectively. While in A the untreated insect shows a four- or five-segmented antenna, B exemplifies the common deformation observed in treated insects, consisting of a lack of flagellum and pedicel segments in the antenna, resulting in only one segment or even no antennae. C represents ×250 magnification in segment IV/V of the untreated insect antenna, showing the amount of trichoid sensilla (Ts), while D demonstrates the effects of CC-EO nanoemulsion in the treated insect by reducing the number of sensilla in the unique antenna segment. E displays the developed Ts distributed per area, and F indicates the undeveloped trichoid sensilla (uTs) and their low distribution per area, both at ×1600 magnification. Observations in experiments indicated that the loss of antenna segments influenced results, such as reduced movement and feeding on treated insects, culminating in the death of all insects with these deformities until the end of the experiments, as well as those that remained in exuvia.

### 3.5. Olfactometer Assays

**Figure 4:**
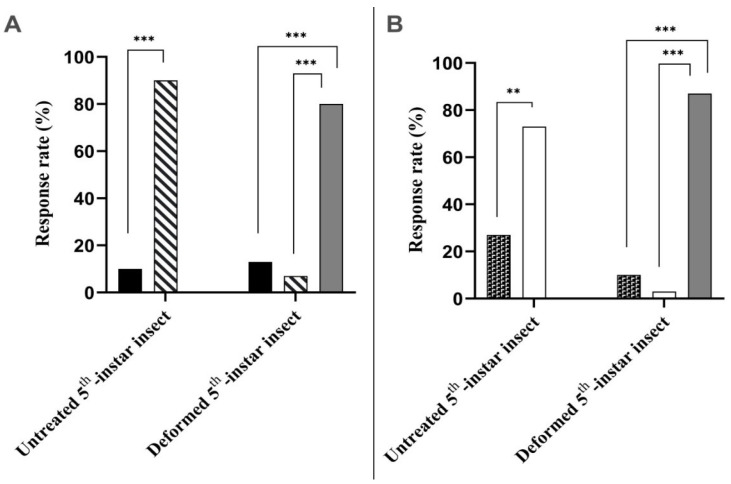
Response rate of *Dysdercus peruvianus* fifth-instar nymphs in olfactometer assays with *Cymbopogon citratus* stimuli. (**A**)—Response of untreated insects with normal antennae and deformed insects with malformed antennae in choice experiments between C. *citratus* essential oil (CC-EO) and liquid paraffin. (**B**)—Response of untreated and treated insects in choice experiments with CC-EO nanoemulsion and blank nanoemulsion. 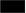 = Pure CC-EO; 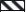 = Liquid paraffin; 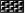 = CC-EO nanoemulsion; 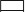 = Blank nanoemulsion; 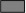 = Non-responsive. Chi-square test: ***—*p* < 0.0001; **—*p* = 0.0003.

Figure 4A shows the response rates of fifth-instar *D. peruvianus* nymphs to CC-EO and liquid paraffin in the Y-tube olfactometer and liquid paraffin in the Y-tube olfactometer. Untreated insects with normal antennae showed a preference for paraffin-treated filter paper, with a response rate of 90% compared with 10% for EO-treated paper (χ^2^ (1) = 38.40, *p* < 0.0001). However, treated insects with deformities in antennae showed a predominant pattern of non-responsive behavior, with 80% of insects remaining in the stem of the tube, while 13.33% chose the EO-treated paper and 6.67% chose the paraffin-treated paper (χ^2^ (1) = 26.79, *p* < 0.0001 for non-responsive × EO analysis; χ^2^ (1) = 32.85, *p* < 0.0001 for non-responsive × paraffin analysis).

In Figure 4B, treatments were performed with a choice between CC-EO nanoemulsion and blank nanoemulsion. Untreated insects predominantly chose the blank nanoemulsion-treated paper, with a response rate of 73.33% of response rate, whereas 26.67% of insects chose the EO nanoemulsion-treated paper (χ^2^ (1) = 13.07, *p* = 0.0003). As observed in EO × paraffin tests, CC-EO nanoemulsion vs. blank nanoemulsion showed a similarly high proportion of non-responsive behavior in deformed insects: 86.67% of individuals were non-responsive, while response rates of 10% and 3.33% were recorded for the EO nanoemulsion and blank nanoemulsion, respectively (χ^2^ (1) = 35.31, *p* < 0.0001 for non-responsive vs. EO nanoemulsion analysis; χ^2^ (1) = 42.09, *p* < 0.0001 for non-responsive vs. blank nanoemulsion analysis).

### 3.6. HS-SPME Analysis

#### Volatile Profiles of *D. peruvianus* and *E. heros*

The volatile organic compound (VOC) profiles of *D. peruvianus* and *E. heros* nymphs were analyzed using HS-SPME/GC-MS, revealing significant qualitative and quantitative differences following treatment with CC-EO nanoemulsion (Table S2).

In untreated *D. peruvianus*, 27 compounds were identified, including terpenes (28.5%), aldehydes (21.0%), and alcohols (10.9%). Major volatiles included 2,9-decanedione (1473.7 ppb), trans-caryophyllene (1153.5 ppb), and 2-octenal (815.4 ppb). Alcohols such as 1-hexanol, 2-octen-1-ol, and 3-octen-1-ol were also present. After nanoemulsion treatment, 26 compounds were detected, with notable compositional shifts: alcohols such as 1-octanol, 1-dodecanol, and phenylethyl alcohol were absent, while new compounds emerged, including 6-methylhept-5-en-2-ol (866.6 ppb) and 5-methyl-2-(1-methylethyl)-phenol (393.3 ppb). Terpenes became the dominant class (59.4%), followed by ketones (12.0%). Trans-caryophyllene increased threefold (3447.7 ppb), and new ketones like 3-methylhexan-2-one (3447.0 ppb) and 2,11-dodecanedione (666.6 ppb) were detected. Several terpenes were exclusive to treated insects, including α-gurjunene, β-elemene, and γ-terpinene, suggesting a substantial effect of the treatment on terpene metabolism.

Untreated *E. heros* emitted 30 compounds, primarily alkanes (47.1%) and ketones (23.6%). Tridecane (19,592.7 ppb), 3-hydroxy-2-butanone (14,102.3 ppb), and 2-octenal (3979.2 ppb) were the most abundant. A single alcohol, 2-octen-1-ol (539.9 ppb), was detected. After treatment, 30 compounds were identified, with aldehydes becoming the major group (36.9%), followed by alkanes (25.4%) and alcohols (16.5%). Seven alcohols were detected in treated insects, including 3-methyl-1-butanol (8706.8 ppb), absent in controls. New esters, such as ethyl isovalerate (4959.3 ppb) and ethyl isobutyrate (1166.4 ppb), also appeared. Aldehyde 2-methylbutanal became the most abundant compound (22,077.9 ppb), while some volatiles such as 2-octen-1-ol, 2-octenal, and 3-hydroxy-2-butanone were no longer detected. These modifications indicate a strong impact of the EO-based nanoemulsion on the metabolic pathways involved in VOC biosynthesis in both species.

Together, the results demonstrate that CC-EO nanoemulsion not only reduces the total number of volatiles in *D. peruvianus* but also shifts the chemical profile toward terpenes and ketones, while in *E. heros*, the treatment induces new volatile pathways, markedly increasing aldehydes and alcohols. These changes may reflect detoxification responses or metabolic disruption induced by the EO components.

## 4. Discussion

### 4.1. CC-EO and Its Nanoemulsion

The CC-EO, which was viscous, yellow, and had a characteristic lemongrass aroma, was obtained and analyzed for its constituents. The EO contained the major monoterpene constituents reported in the literature, along with two oxidation isomers of linalool [44]. Regarding the EO yield from fresh leaves of *C. citratus*, the present study reported 0.39%, which falls within the expected range for hydrodistillation using a Clevenger-type apparatus. Typically, yields reported in the literature for CC-EO extracted from fresh leaves vary from 0.3% to 1.0%, depending on factors such as plant part, moisture content, and distillation conditions. Notably, Anggraeni et al. reported a lower yield of 0.24% from dried stem material (1272 g) using steam distillation in a Karlsruhe apparatus [45].

The major compound characterized by GC-MS analysis was citral, a mixture of the isomers geranial and neral, accounting for 59.52% of the CC-EO total composition. Geranial and neral are commonly reported as major components of CC-EO in several studies, with different proportions; comparisons with the literature show that the area percentage values of both isomers approach those reported in this study, with EO composition ranging from 25–35% [46,47,48,49,50]. Citral is a key monoterpenoid aldehyde with documented antimicrobial, antifungal, and insecticidal properties, often associated with the biological efficacy of CC-EO [51,52,53]. The simultaneous presence of linalool oxide isomers indicates a significant proportion of oxygenated monoterpenes. This chemical profile underscores the predominance of oxygenated constituents, which are known for enhancing the bioavailability and biological effects of EOs [54].

Citral, the predominant constituent of the EO evaluated in this study, may be the main agent responsible for the observed insecticidal activity. However, the biological effects may also arise from synergistic interactions among the oxygenated compounds present in the CC-EO. This hypothesis underscores the need for further targeted studies to elucidate the specific mechanisms of action of CC-EO and its metabolites on the developmental processes of *E. heros* and *D. peruvianus*.

Nanoemulsions are mixtures of two immiscible liquid phases with a dispersed phase particle size range of 20–500 nm [27]. Previous studies have reported *C. citratus* nanoemulsions ranging from 20 to 200 nm [49,50,55,56]. Formulations of CC-EO above 200 nm were also reported [57,58,59]. The data from the present study indicate that the formulation with an HLB value of 14 yielded droplet sizes ranging from 200 to 350 nm over the evaluated time interval. A nanoemulsion is typically destabilized by Ostwald ripening, leading to an increase in EO droplet size over a 90-day particle-size monitoring period [60]. However, no signs of coalescence-related phenomena, such as creaming or phase separation, were observed in the EO nanoemulsion throughout the 90-day evaluation period [27]. The nanoemulsion with an HLB value of 14 exhibited a PDI below 0.3, indicating a monodisperse droplet distribution. Zeta potential measurements ranged from −20 to −30 mV, reflecting electrostatic stability and suitable physicochemical parameters for a stable formulation [61].

### 4.2. Insecticidal Activity

The present study demonstrated the biotechnological potential of CC-EO and its nanoemulsion-based formulation, as evidenced by their insecticidal activity against the stink bugs *E. heros* and *D. peruvianus*. CC-EO has shown insecticidal activity against diverse insects—from cotton pests and stored-product beetles to disease vectors such as mosquitoes [62,63,64,65].

Emulsified CC-EO formulations have achieved near-total mortality in various insect species; for example, 100% of *Musca domestica* larvae were killed within 48 h, and ~98% mortality of *Tribolium castaneum* adults was achieved within 72 h at low EO concentrations [66,67]. Likewise, a Tween 80-stabilized CC-EO nanoemulsion caused ~96–100% mortality of *Anticarsia gemmatalis* caterpillars within 48 h [68].

Previous studies using Lauraceae-derived EO nanoemulsions on *D. peruvianus* recorded much higher mortalities than those observed here—for example, *Ocotea elegans* EO caused ~90% mortality (only ~10% survival) and *Persea venosa* EO achieved ~55% mortality [40,69]. By comparison, the CC-EO nanoemulsion in this study produced a more moderate, yet statistically significant, 33.3% mortality in *D. peruvianus* via topical application. This reduced efficacy may reflect the action of detoxification enzymes common in hemipterans (e.g., cytochrome P450s, glutathione S-transferases, esterases) that can metabolize EO constituents, thereby reducing acute toxicity [70].

Recent biochemical and physiological studies with CC-EOs support this interpretation. In the mango spider mite *Oligonychus mangiferus*, exposure of adult females to LC_50_ levels of lemongrass and peppermint EOs caused a significant increase in antioxidant enzymes (peroxidase and catalase). Simultaneously, the detoxifying enzymes α-esterase, b-esterase, and glutathione S-transferase were inhibited, indicating that detoxification and oxidative-stress pathways are primary targets of these oils [71]. In the noctuid *Agrotis ipsilon*, sublethal concentrations of CC-EO reduced larval survival, altered larval and pupal development and adult emergence, and significantly modified carboxylesterase (α- and β-esterase) and glutathione S-transferase activities together with oxidative-stress markers [72]. In *Aedes aegypti*, a citral-rich *Cymbopogon flexuosus* EO modulated detoxification enzymes, including non-specific esterases, glutathione S-transferase, and mixed-function oxidases, and produced a partial inhibition (≈12–34%) of acetylcholinesterase activity in larval and adult homogenates [73]. Metabolism studies in *Trichoplusia ni* larvae further showed that citral is rapidly biotransformed to geranic and neric acids, and that co-application of enzyme inhibitors, such as piperonyl butoxide, can enhance citral toxicity even though its metabolic profile remains largely unchanged [74].

In addition to their role in metabolic detoxification, plant EOs and their monoterpenes, including citral, are increasingly recognized as neuroactive compounds. Reviews on EO-based insecticides highlight inhibition of acetylcholinesterase, interference with cholinergic, GABAergic, and octopaminergic pathways, and effects on ion-transport enzymes such as Na^+^/K^+^-ATPase as major neurophysiological targets in insects [75,76,77,78].

Taken together, these data are consistent with the lethargic, moribund phenotypes recorded here and support the view that CC-EO and its nanoemulsion act through combined metabolic and neurophysiological disruption rather than purely mechanical or cuticular effects.

In contrast, contact exposure to the CC-EO nanoemulsion resulted in 86.7% mortality in *D. peruvianus*, likely due to prolonged residual action on treated surfaces that enhances cuticular absorption of the EO and overall toxic load [78].

EOs from other plant species (e.g., *Piper aduncum*) have shown insecticidal activity against *E. heros* [79,80]. However, to our knowledge, no prior work has examined an EO-based nanoemulsion on this pest. This study provides the first evidence of an insecticidal effect of a CC-EO nanoemulsion against *E. heros*, achieving considerable adult mortality in both topical (~53%) and contact (~83%) exposure assays. We also observed strong ovicidal activity in both *E. heros* and *D. peruvianus*, manifested as significant inhibition of egg hatching after treatment with either the pure EO or its nanoemulsion. This ovicidal activity likely stems from the ability of nanoemulsion components to penetrate the chorionic egg membrane and disrupt embryogenesis [81,82].

Contact with EO-treated surfaces can induce physicochemical interactions with insect antennal sensilla, leading to structural damage [41]. Consistent with this, our SEM analysis revealed a reduction in the number of trichoid sensilla and a loss of antennal segmentation in both exposed species. Nanoparticles in the CC-EO nanoemulsion likely adhered to sensillar surfaces, causing progressive sensilla degradation and necrosis and thereby diminishing the functional antennal area [64,83]. Exposure to plant-derived compounds is known to induce cuticle and appendage deformities in insects. For example, treatments with certain plant EOs have been associated with missing antennal segments, deformed legs, and wing malformations in hemipteran insects [84,85,86,87]. Antennal damage incurred during nymphal stages has limited regenerative capacity [88]. This likely explains the antennal anomalies observed in fourth-instar *E. heros* and *D. peruvianus* after exposure to the nanoemulsion.

In contact assays, *D. peruvianus* exhibited a higher incidence of antennal deformities than *E. heros*, suggesting greater susceptibility to the EO’s sublethal effects. Moreover, *D. peruvianus* also exhibited more active locomotion than *E. heros* during exposure, which could increase cuticular uptake of the nanoemulsion and thus amplify its toxic effects [89]. In addition, grooming behavior may contribute to the observed antennal damage. Frequent rubbing of the antennae with their forelegs can cause mechanical abrasion or transfer of irritant residues onto the sensilla. Over time, such autogrooming might lead to segmental wear and sensillar degradation. Olfactometric assays further revealed that *D. peruvianus* individuals with antennal malformations had impaired olfaction and reduced responsiveness in a Y-tube setup, whereas untreated insects with intact antennae showed strong avoidance of odor sources containing CC-EO or its nanoemulsion. This confirms the repellent activity of lemongrass-based formulations, consistent with reports that CC-EO exhibits high repellency against stored-product pests [90].

Moribund insects often exhibit lethargy, which may, in part, arise from antennal damage that hinders the detection of food and water cues. However, lethal outcomes of EO exposure are more directly tied to neurotoxic and endocrine-disrupting mechanisms. Many terpene compounds in EOs act on the insect nervous system—for example, by antagonizing GABA receptors or disrupting octopaminergic signaling—and some function as Insect Growth Regulators (IGRs) that alter levels of juvenile hormone and ecdysteroids [91,92,93,94].

In our study, triflumuron caused high nymphal mortality and numerous deformities, especially under topical treatment (Table S1). Triflumuron acts as a chitin synthesis inhibitor and IGR, disrupting development even at low doses [95]. Benzoylphenyl ureas (BPUs), a class of IGRs, are generally considered safer for the environment than earlier classes of insecticides (e.g., organochlorines, organophosphates) due to lower persistence and faster photodegradation [96]. However, some BPU compounds still pose ecological risks, showing high acute toxicity to fish and aquatic invertebrates and moderate toxicity to pollinators and earthworms [97]. Notably, the poor water solubility of triflumuron underscores the benefit of nanoemulsion formulations for improving the delivery of hydrophobic active ingredients and reducing the need for organic solvents [98,99]. In direct comparison, topical application of triflumuron produced higher mortality and more pronounced sublethal effects in both *E. heros* and *D. peruvianus* than the CC-EO nanoemulsion, reflecting the potent IGR mode of action of the BPU. Conversely, under contact (residual) exposure, the CC-EO nanoemulsion outperformed triflumuron in terms of efficacy, likely because its residual activity prolongs insect exposure to its bioactive compounds [100].

Our results are consistent with the established efficacy of triflumuron and highlight the potential of the CC-EO nanoemulsion. Notably, this formulation contained only 5% EO yet achieved substantial insecticidal and ovicidal effects. These findings warrant further toxicological studies on *E. heros* and *D. peruvianus* (e.g., determining lethal dose thresholds), which will be important for assessing the integration of lemongrass EO-based formulations into integrated pest management programs against these pests.

### 4.3. HS-SPME Analysis

HS-SPME analysis revealed that *E. heros* exhibits distinct volatile profiles before and after exposure to the CC-EO nanoemulsion. In untreated insects, the emissions included hexanoic and 2-octenoic acids, dodecane, tridecane, tetradecane, dodecanol, and (E)-2-octenal, all of which are commonly associated with chemical defense [101]. Among them, 2-octenoic acid has a known semiochemical role in *Ceratitis capitata* and *Cimex hemipterus* [102,103]. Acetoin (14,102.3 ppb), a bacterial metabolite produced by species such as *Bacillus subtilis*, was also detected at high levels, suggesting involvement in stress signaling or insect–plant interaction [104,105,106].

After treatment, short-chain volatiles absent in controls showed notable increases: 2-methylbutanal (22,077.9 ppb) and 3-methylbutanol (8706.8 ppb). Tridecane remained consistently high (17,264.4 ppb). The appearance of isovaleraldehyde is consistent with stress-related glandular emissions previously described in heteropterans [107]. Compounds like 2-methylbutanoic acid and 3-methylbutanol have been linked to alarm and aggregation responses [108,109]. The emission of these volatiles indicates stress-induced modulation, while the persistent presence of tridecane suggests a constitutive defense function [101].

In *D. peruvianus*, untreated individuals exhibited a diverse blend of volatiles dominated by 2,9-decanedione (1473.7 ppb), trans-caryophyllene (1153.5 ppb), and (E)-2-octenal (815.4 ppb). In other systems, these compounds have played roles in aggregation, sexual signaling, or alarm communication [102,110,111]. 2,9-decanedione has been proposed as a marker of metabolic stress in social insects [112].

Treatment with CC-EO nanoemulsion led to a reduction in the number of detected volatiles (29 → 26) and a marked shift in profile. Alcohols that mediate insect communication, such as 1-octanol, 1-hexanol, and 2-phenethyl alcohol, were no longer detected [113,114]. Conversely, 6-methylhept-5-en-2-ol (866.6 ppb), an aggregation-sex pheromone in beetles, appeared exclusively in treated insects [115,116].

Trans-caryophyllene increased significantly from 1153.5 to 3447.7 ppb, while alpha-humulene declined. Newly detected sesquiterpenes such as limonene, alpha-copaene, and beta-selinene indicate activation of terpene biosynthetic pathways [117]. Geranic acid was detected at 1057.1 ppb and is likely due to the enzymatic oxidation of citral isomers in the CC-EO nanoemulsion, suggesting active metabolism of exogenous constituents [74].

In *D. peruvianus*, these changes coincided with antennal deformities and reduced responsiveness in Y-tube assays, suggesting a potential link between morphological damage and disrupted semiochemical communication [118]. In Y-tube olfactometers, untreated individuals with intact antennae strongly discriminated against odor sources. Approximately 90% avoided CC-EO in favor of liquid paraffin, and 73.33% preferred the blank nanoemulsion over CC-EO nanoemulsion, a pattern consistent with repellent perception of EO mixtures and formulations by functional chemosensory systems [119].

In sharp contrast, treated insects with antennal malformations displayed nonresponsive behavior, with 80–86.67% remaining immobile in the Y-tube stem. When responses occurred, choices were random, 13.33% versus 6.67% for EO versus paraffin, and 10% versus 3.33% for EO nanoemulsion vs. blank. This behavioral collapse correlates with concurrent shifts in the VOC profile documented by HS-SPME, a marked reduction in trichoid sensilla density visualized by scanning electron microscopy, and the appearance of atypical volatiles such as sulcatol, previously reported as an aggregation pheromone in beetles, which may interfere with olfactory processing [115,116]. In summary, both species exhibit chemical modulation after exposure to CC-EO nanoemulsion; morphological effects are more pronounced in *D. peruvianus*, for which behavioral impairment was demonstrated. In *E. heros*, chemical changes were evident, and antennal alterations occurred at a lower frequency, but the available dataset does not support behavioral inference. These outcomes highlight species-specific sensitivity and support targeted follow-up to examine how antennal integrity relates to VOC emission and chemosensory function in heteropterans.

## 5. Conclusions

This study demonstrates the biotechnological potential of *Cymbopogon citratus* essential oil (CC-EO) and its nanoemulsion as candidate biopesticides against the hemipteran pests *Euschistus heros* and *Dysdercus peruvianus*. The nanoemulsion, containing only 5% EO, caused substantial nymphal and adult mortality under topical and, especially, residual contact exposure, and exhibited marked ovicidal activity, completely inhibiting egg hatching when the pure EO was used. In addition, contact exposure to CC-EO nanoemulsion induced antennal malformations and reduced trichoid sensilla density in both species, and, in *D. peruvianus*, these structural alterations were associated with loss of odor-guided behavior in Y-tube assays and pronounced shifts in semiochemical emission profiles. Together, these lethal and sublethal effects indicate a multi-target mode of action involving contact toxicity, disruption of chemosensory structures and functions, and modulation of stress-related volatile emissions.

The insecticidal and ovicidal efficacy observed, combined with the 90-day physicochemical stability of the nanoemulsion, supports its consideration as a biorational alternative to conventional synthetic insecticides in soybean and cotton IPM programs. Sublethal impacts on chemoreception and host-location behavior suggest that, beyond direct mortality, CC-EO nanoemulsions may contribute to pest suppression through multiple pathways by impairing host finding and semiochemical communication, a hypothesis that merits further population-level and field validation. Methodologically, this work also highlights the value of combining SEM, behavioral olfactometry, and HS-SPME/GC-MS to diagnose both lethal and sublethal responses to essential oil-based formulations in hemipteran pests, paving the way for future studies on field persistence, selectivity to natural enemies, and integration with semiochemical-based monitoring tools.

## Figures and Tables

**Table 1:** Formulations of *Cymbopogon citratus* essential oil nanoemulsion prepared for the study, with their respective hydrophilic–lipophilic balance (HLB) values and constituents (values in mg).

Materials	HLB 15 (A)	HLB 15 (B)	HLB 14	HLB 13
Tween 20	86.00	-	-	-
Tween 80	-	100.00	90.65	81.30
Span 80	14.00	0	9.35	18.70
Essential oil	100.00	100.00	100.00	100.00
Mili-Q water	1800	1800	1800	1800

**Table 2:** Mean percentage of *Euschistus heros* and *Dysdercus peruvianus* nymph eclosion rate from treated eggs with *Cymbopogon citratus* essential oil (CC-EO), EO nanoemulsion, blank nanoemulsion (without EO), positive control (triflumuron), and their respective untreated control after 10 days of contact treatment. Data were analyzed by ANOVA and Tukey’s test (*n* = 20 eggs in 9 replicates per treatment).

Treatment × *Dysdercus*	Eclosion Rate (Mean % ± SD)	*p* Value
Pure CC-EO	0	<0.0001
CC-EO nanoemulsion	15.00 ± 2.45	<0.0001
Blank nanoemulsion	70.0 ± 3.70	0.6496
Untreated control	78.35 ± 3.20	-
Positive control (triflumuron)	12.75 ± 1.81	<0.0001
ANOVA	*F*(4,40) = 70.61	
**Treatment × *Euschistus***	**Eclosion Rate (Mean % ± SD)**	***p* Value**
Pure CC-EO	0	<0.0001
CC-EO nanoemulsion	36.10 ± 3.53	<0.0001
Blank nanoemulsion	75.00 ± 2.70	0.5312
Untreated control	83.35 ± 2.24	-
Positive control (triflumuron)	18.35 ± 1.00	<0.0001
ANOVA	*F*(4,40) = 90.28	

## Data Availability

The original contributions presented in this study are included in the article/Supplementary Materials. Further inquiries can be directed to the corresponding authors.

## Supplementary Materials

The following supporting information can be downloaded at https://www.mdpi.com/article/10.3390/insects16121254/s1, Figure S1: HLB 14 nanoemulsion characterization. Particle size distribution: (A) *Cymbopogon citratus* essential oil nanoemulsion (242 nm) and (B) blank nanoemulsion without essential oil (195.11 nm). Zeta potential distribution: (C) essential oil nanoemulsion (−22.1 mV) and (D) blank nanoemulsion (−9.6 mV). Table S1: Percentage of deformations observed in *Dysdercus peruvianus* and *Euschistus heros* insects after topical and contact treatments with *Cymbopogon citratus* essential oil, oil nanoemulsion, blank nanoemulsion (without essential oil), positive control (triflumuron), and their respective untreated control. Table S2: Concentrations of volatile compounds (ppb) detected in *Euschistus heros* and *Dysdercus peruvianus* by HS-SPME/GC-MS analysis. Data from untreated control samples and samples treated with *Cymbopogon citratus* essential oil nanoemulsion.

## Abbreviations

The following abbreviations are used in this manuscript:

EO	Essential oil
CC-EO	*Cymbopogon citratus* essential oil
HS-SPME	Headspace solid-phase microextraction
IPM	Integrated Pest Management
HLB	Hydrophilic–Lipophilic Balance
PDI	Polydispersity Index
SISGEN	National System for the Management of Genetic Heritage and Associated Traditional Knowledge
DAT	Days after treatment
GC-MS	Gas Chromatography coupled to Mass Spectrometry
SEM	Scanning Electron Microscopy
Ts	Trichoid sensilla
uTs	Undeveloped trichoid sensilla
VOC	Volatile Organic Compounds

## References

[1] Alston J.M., Pardey P.G. (2014). Agriculture in global economy. J. Econom. Perspect..

[2] FAO (2022). Agricultural Production Statistics: 2000–2021.

[3] Toplicean I.M., Datcu A.D. (2024). An overview on Bioeconomy in agricultural sector, biomass production, recycling methods, and circular economy considerations. Agriculture.

[4] OECD-FAO (2023). Agricultural Outlook 2023–2032.

[5] Fuglie K.O., Morgan S., Jelliffe J. (2024). World Agricultural Production, Resource Use, and Productivity, 1961 2020.

[6] FAO FAOSTAT 2024. https://www.fao.org/faostat/en/#data/QCL/visualize.

[7] Silva M.L., Silva S.X.B., Sugayama R.L., Rangel L.E.P., Ribeiro L.C., Sugayama R.L., Silva M.L., Silva S.X.B., Ribeiro L.C., Rangel L.E.P. (2015). Defesa vegetal: Conceitos, escopo e importância estratégica. Defesa Vegetal—Fundamentos, Ferramentas, Políticas e Perspectivas.

[8] Bueno A.F., Panizzi A.R., Hunt T.E., Dourado P.M., Pitta R.M., Gonçalves J. (2021). Challenges for adoption of Integrated Pest Management (IPM): The soybean example. Neotrop. Entomol..

[9] FAO New Standards to Curb the Global Spread of Plant Pests and Diseases 2019. https://www.fao.org/newsroom/detail/New-standards-to-curb-the-global-spread-of-plant-pests-and-diseases/en.

[10] Oliveira C.M., Auad A.M., Mendes S.M., Frizzas M.R. (2014). Crop losses and the economic impact of insect pests on Brazilian Agriculture. Crop Prot..

[11] Forero D., Castro-Huertas V., Morales-Devia H., Barão K.R., Bianchi F.M., Campos L.A., Dellapé P.M., Melo M.C., Schwertner C.F. (2024). Heteroptera research in Latin America and the Caribbean (Insecta, Hemiptera): Status and perspectives in the 21st century. An. Acad. Bras. Ciênc..

[12] Corrêa-Ferreira B.S., Sosa-Gómez D.R. (2017). Percevejos e o Sistema de Produção Soja-Milho.

[13] Belluco S., Bertola M., Montarsi F., Di Martino G., Granato A., Stella R., Martinello M., Bordin F., Mutinelli F. (2023). Insects and public health: An overview. Insects.

[14] Panizzi A.R., Lucini T.L. (2022). The overlooked role of weed plants affecting pest stink bug (Hemiptera: Heteroptera: Pentatomidae) bioecology in the Neotropics. Arthropod-Plant Interact..

[15] Rodrigues L.M., Garcia A.G., Parra J.R.P. (2023). Ecological zoning of *Euschistus heros* in Brazil based on the net reproductive rate at different temperatures and relative-humidity levels. J. Econom. Entomol..

[16] Rosado H.C., Anholeti M.C., Santos M.G., Santos-Mallet J.R., Figueiredo M.R., Mello C.B., Gonzalez M.S., Paiva S.R., Feder D. (2019). Effects of semi-purified fractions from stems of *Clusia hilariana* on the development of *Dysdercus peruvianus*. Rev. Bras. Farmacogn..

[17] FAO (2024). Pesticides Use and Trade—1990–2022.

[18] Tibola C.M., Silva L., Sgubin F., Omoto C. (2021). Monitoring resistance of *Euschistus heros* (Fabricius) (Hemiptera: Pentatomidae) to insecticides by using encapsulated artificial diet bioassay. Insects.

[19] Betinelli P.A., Corrêa F.R., da Silva N.F., Cavalcante W.S.S., Ribeiro D.F., Rodrigues E. (2023). Sinergismo na combinação de (acefato + bifentrina + acetamiprido) no controle do percevejo-marrom. Braz. J. Sci..

[20] Wahocho H.J., Murtaza A., Otho S.A., Kubar M.I., Khoso N., Chand K., Mangi S. (2023). Performance of different botanicals against red cotton bug, *Dysdercus cingulatus* (Fab.) under laboratory conditions. J. Agric. Rural. Stud..

[21] Phogat A., Singh J., Kumar V., Malik V. (2022). Toxicity of the acetamiprid insecticide for mammals: A review. Environ. Chem. Lett..

[22] Mota T.F.M., Oliveira W.L., Gonçalves S., Vasconcelos M.W., Miglioranza K.S.B., Ghisi N.C. (2023). Are the issues involving acephate already resolved? A scientometric review. Environ. Res..

[23] Wei F., Cheng F., Li H., You J. (2024). Imidacloprid affects human cells through mitochondrial dysfunction and oxidative stress. Sci. Total Environ..

[24] George D.R., Finn R.D., Graham K.M., Sparagano O.A.E. (2014). Present and future potential of plant-derived products to control arthropods of veterinary and medical significance. Parasit. Vectors.

[25] Ataei P., Gholamrezai S., Movahedi R., Aliabadi V. (2021). An analysis of farmers’ intention to use green pesticides: The application of the extended theory of planned behavior and health belief model. J. Rural. Stud..

[26] Mukarram M., Khan M.M.A., Zehra A., Choudhary S., Naeem M., Aftab T., Aftab T., Hakeem K.R. (2021). Biosynthesis of lemongrass essential oil and the underlying mechanism for its insecticidal activity. Medicinal and Aromatic Plants—Healthcare and Industrial Applications.

[27] Kumar A., Kanwar R., Mehta S.K. (2025). Nanoemulsion as an effective delivery vehicle for essential oils: Properties, formulation methods, destabilizing mechanisms and applications in agri-food sector. Next Nanotechnol..

[28] Singh I.R., Pulikkal A.K. (2022). Preparation, stability and biological activity of essential oil-based nano emulsions: A comprehensive review. Open Nano.

[29] Mota A.H., Sousa A., Figueira M., Amaral M., Sousa B., Rocha J., Fattal E., Almeida A.J., Reis C.P., Hussain C.M. (2020). Natural-based consumer health nanoproducts: Medicines, cosmetics, and food supplements. Handbook of Functionalized Nanomaterials for Industrial Applications.

[30] Maurya A., Singh V.K., Das S., Prasad J., Kedia A., Upadhyay N., Dubey N.K., Dwivedy A.K. (2021). Essential oil nanoemulsion as eco-friendly and safe preservative: Bioefficacy against microbial food deterioration and toxin secretion, mode of action, and future opportunities. Front. Microbiol..

[31] Pires P.C., Fernandes M., Nina F., Gama F., Gomes M.F., Rodrigues L.E., Meirinho S., Silvestre S., Alves G., Santos A.O. (2023). Innovative aqueous nanoemulsion prepared by phase inversion emulsification with exceptional homogeneity. Pharmaceutics.

[32] Soreng R.J., Peterson P.M., Zuloaga F.O., Romaschenko K., Clark L.G., Teisher J.K., Gillespie L.J., Barberá P., Welker C.A.D., Kellogg E.A. (2022). A worldwide phylogenetic classification of the Poaceae (Gramineae) III: An update. J. Sys. Evol..

[33] Almeida K.B., Araujo J.L., Cavalcanti J.F., Romanos M.T.V., Mourão S.C., Amaral A.C.F., Falcão D.Q. (2018). In vitro release and anti-herpetic activity of *Cymbopogon citratus* volatile oil-loaded nanogel. Rev. Bras. Farmacogn..

[34] Cortes-Torres A.G., López-Castillo G.N., Marín-Torres J.L., Portillo-Reyes R., Luna F., Baca B.E., Sandoval-Ramírez J., Carrasco-Carballo A. (2023). *Cymbopogon citratus* essential oil: Extraction, GC–MS, phytochemical analysis, antioxidant activity, and in silico molecular docking for protein targets related to CNS. Curr. Issues Mol. Biol..

[35] Dangol S., Poudel D.K., Ojha P.K., Maharjan S., Poudel A., Satyal R., Rokaya A., Timsina S., Dosoky N.S., Satyal P. (2023). Essential oil composition analysis of *Cymbopogon* species from Eastern Nepal by GC-MS and chiral GC-MS, and antimicrobial activity of some major compounds. Molecules.

[36] Ostertag F., Weiss J., McClements D.J. (2012). Low-energy formation of edible nanoemulsions: Factors influencing droplet size produced by emulsion phase inversion. J. Colloid. Interface Sci..

[37] Mourão A.P.M., Panizzi A.R. (2002). Photophase influence on the reproductive diapause, seasonal morphs, and feeding activity of *Euschistus heros* (FABR., 1798) (Hemiptera: Pentatomidae). Braz. J. Biol..

[38] Gonzalez M.S., Lima B.G., Oliveira A.F.R., Nunes D.D., Fernandes C.P.F., Santos M.G., Tietbohl L.A.C., Mello C.B., Rocha L., Feder D. (2014). Effects of essential oil from eaves of *Eugenia sulcata* on the development of agricultural pest insects. Rev. Bras. de Farmacogn..

[39] Fernandes C.P., Almeida F.B., Silveira A.N., Gonzalez M.S., Mello C.B., Feder D., Apolinário R., Santos M.G., Carvalho J.C.T., Tietbohl L.A.C. (2014). Development of an insecticidal nanoemulsion with *Manilkara subsericea* (Sapotaceae) extract. J. Nanobiotechnology.

[40] Esteves R.S., Apolinário R., Machado F.P., Folly D., Viana V.C.R., Soares A.P., Jumbo L.O.V., Svacina T., Santos M.G., Ricci-Junior E. (2023). Insecticidal activity evaluation of *Persea venosa* Nees & Mart. essential oil and its nanoemulsion against the cotton stainer bug *Dysdercus peruvianus* (Hemiptera: Pyrrhocoridae) and pollinator bees. Ind. Crops Prod..

[41] Callahan P.S. (1975). Insect antennae with special reference to the mechanism of scent detection and the evolution of the sensilla. Int. J. Insect Morph. Embriol..

[42] Biasazin T.D., Chernet H.T., Herrera S.L., Bengtsson M., Karlsson M.F., Lemmen-Lechelt J.K., Dekker T. (2018). Detection of volatile constituents from food lures by tephritid fruit flies. Insects.

[43] Adams R.P. (2007). Identification of Essential Oil Components by Gas Chromatography/Mass Spectrometry.

[44] Sforcin J.M., Amaral J.T., Fernandes A.J., Sousa J.P.B., Bastos J.K. (2009). Lemongrass effects on IL-1β and IL-6 production by macrophages. Nat. Prod. Res..

[45] Anggraeni N.I., Hidayat I.W., Rachman S.D., Ersanda (2018). Bioactivity of essential oil from lemongrass (*Cymbopogon citratus* Stapf) as antioxidant agent. AIP Conf. Proc..

[46] Olivero-Verbel J., Nerio L.S., Stashenko E.E. (2009). Bioactivity against *Tribolium castaneum* Herbst (Coleoptera: Tenebrionidae) of *Cymbopogon citratus* and *Eucalyptus citriodora* essential oils grown in Colombia. Pest Manag. Sci..

[47] Ntonga P.A., Baldovini N., Mouray E., Mambu L., Belong P., Grellier P. (2014). Activity of *Ocimum basilicum*, *Ocimum canum*, and *Cymbopogon citratus* essential oils against *Plasmodium falciparum* and mature-stage larvae of *Anopheles funestus* s.s. Parasite.

[48] Vera S.S., Zambrano D.F., Méndez-Sanchez S.C., Rodríguez-Sanabria F., Stashenko E.E., Luna J.E.D. (2014). Essential oils with insecticidal activity against larvae of *Aedes aegypti* (Diptera: Culicidae). Parasitol. Res..

[49] Gago C., Antão R., Dores C., Guerreiro A., Miguel M.G., Faleiro M.L., Figueiredo A.C., Antunes M.D. (2020). The effect of nanocoatings enriched with essential oils on ‘Rocha’ pear long storage. Foods.

[50] Boudechicha A., Aouf A., Farouk A., Ali H.S., Elkhadragy M.F., Yehia H.M., Badr A.N. (2023). Microfluidizing technique application for Algerian *Cymbopogon citratus* (DC.) Stapf effects enhanced volatile content, antimicrobial, and anti-mycotoxigenic properties. Molecules.

[51] Habib S., Gupta P., Bhat S.S., Gupta J. (2021). In silico, in-vitro and in vivo screening of biological activities of citral. Int. J. Vitam. Nutr. Res..

[52] Jin C., Han H., Xie Y., Li B., Zhang Z., Zhang D. (2022). Toxicity, behavioral effects, and chitin structural chemistry of *Reticulitermes flaviceps* exposed to *Cymbopogon citratus* EO and its major constituent citral. Insects.

[53] Gutiérrez-Pacheco M.M., Torres-Moreno H., Flores-Lopez M.L., Guadarrama N.V., Ayala-Zavala J.F., Ortega-Ramírez L.A., López-Romero J.C. (2023). Mechanisms and applications of citral’s antimicrobial properties in food preservation and pharmaceuticals formulations. Antibiotics.

[54] De Sousa D.P., Damasceno R.O.S., Amorati R., Elshabrawy H.A., De Castro R.D., Bezerra D.P., Nunes V.R.V., Gomes R.C., Lima T.C. (2023). Essential oils: Chemistry and pharmacological activities. Biomolecules.

[55] Juniatik M., Hidayati K., Wulandari F.P., Pangestuti N., Munawaroh N., Martien R., Utami S. (2017). Formulation of nanoemulsion mouthwash combination of lemongrass oil (*Cymbopogon citratus*) and kaffir lime oil (*Citrus hystrix*) against *Candida albicans* ATCC 10231. Trad. Med. J..

[56] Marinkovic J., Nikolic B., Markovic T., Radunovic M., Ilic J., Boskovic M., Ciric A., Markovic D. (2021). *Cymbopogon citratus* essential oil: An active principle of nanoemulsion against *Enterococcus faecalis* root canal biofilm. Future Microbiol..

[57] Bonferoni M.C., Sandri G., Rossi S., Usai D., Liakos I., Garzoni A., Fiamma M., Zanetti S., Athanassiou A., Caramella C. (2017). A novel ionic amphiphilic chitosan derivative as a stabilizer of nanoemulsions: Improvement of antimicrobial activity of *Cymbopogon citratus* essential oil. Colloids Surf. B Biointerfaces.

[58] Macedo I.T.F., Oliveira L.M.B., André W.P.P., Filho J.V.A., Santos J.M.L., Rondon F.C.M., Ribeiro W.L.C., Camurça-Vasconcelos A.L.F., Oliveira E.F., Paula H.C.B. (2019). Anthelmintic effect of *Cymbopogon citratus* essential oil and its nanoemulsion on sheep gastrointestinal nematodes. Braz. J. Vet. Parasitol..

[59] Bezerra D.C., Oliveira A.E.M.F.M., Silva L.E., Amaral W., Nascimento Y.M., Tavares J.F., Machado F.P., Fernandes C.P. (2023). Surfactant-free nano-emulsions from two lemongrass essential oils: Investigation of temperature ramp influence. Food Chem. Adv..

[60] Kim J., Noh Y., McClements D.J., Choi S.J. (2024). Impact of hydrophilic substances on Ostwald ripening in emulsions stabilized by varied hydrophilic group surfactants. NPJ Sci. Food.

[61] Iqbal N., Hazra D.K., Purkait A., Agrawal A., Kumar J. (2022). Bioengineering of neem nano-formulation with adjuvant for better adhesion over applied surface to give long term insect control. Colloids Surf. B Biointerfaces.

[62] Pushpanathan T., Jebanesan A., Govindarajan M. (2006). Larvicidal, ovicidal and repellent activities of *Cymbopogan citratus* Stapf (Graminae) essential oil against the filarial mosquito *Culex quinquefasciatus* (Say) (Diptera: Culicidae). Trop. Biomed..

[63] Bumbulyte G., Budiene J., Buda V. (2023). Essential oils and their components control behaviour of yellow mealworm (*Tenebrio molitor*) larvae. Insects.

[64] Kobenan K.C., Bini K.K.N., Kouakou M., Kouadio I.S., Zengin G., Ochou G.E.C., Boka N.N.K., Menozzi P., Ochou O.G., Dick A.E. (2021). Chemical composition and spectrum of insecticidal activity of the essential oils of *Ocimum gratissimum* L. and *Cymbopogon citratus* Stapf on the main insects of the cotton entomofauna in Côte d’Ivoire. Chem. Biodiv..

[65] Radunz A.L., Radunz M., Bizollo A.R., Tramontin M.A., Radunz L.L., Mariot M.P., Tempel-Stumpf E.R., Calisto J.F.F., Zaniol F., Albeny-Simões D. (2024). Insecticidal and repellent activity of native and exotic lemongrass on maize weevil. Braz. J. Biol..

[66] Kumar P., Mishra S., Malik A., Satya S. (2011). Repellent, larvicidal and pupicidal properties of essential oils and their formulations against the housefly, *Musca domestica*. Med. Vet. Entomol..

[67] Tanveer M., Ejaz S., Zaka S.M., Batool M., Zahra T., Saghir M., Saeed Q. (2022). Toxicology of diatomaceous earth, phyto oils and their admixed emulsions against adults of *Tribolium castaneum* (Herbst). Toxicol. Rep..

[68] Vicenço C.B., Silvestre W., Pauletti G.F. (2023). Insecticidal activity of lemongrass essential oil and its major compounds on velvet caterpillar. Pesqu. Agropec. Gaúcha.

[69] Nascimento L., Apolinário R., Machado F.P., Correa A.L., Caldas G.R., Ruppelt B.M., Souza K.F.C., Gouveia G., Burth P., Falcao D.Q. (2020). Effects of nanoemulsion and essential oil from the leaves of *Ocotea elegans* against *Dysdercus peruvianus*. Res. Soc. Dev..

[70] Ullah R.M.K., Gao F., Sikandar A., Wu H. (2023). Insights into the effects of insecticides on aphids (Hemiptera: Aphididae): Resistance mechanisms and molecular basis. Int. J. Mol. Sci..

[71] Ahmed M.M., Abdelwines M.A. (2024). Toxicological and physiological activity of lemongrass and peppermint essential oils as acaricidal agents on life-table parameters of *Oligonychus mangiferus* (Rahman & Sapra) and its predatory mite, *Cydnoseius negevi* (Swirskii & Amitai). Phytoparasitica.

[72] Moustafa M.A., Shehata M.G., Abou-El-Ela R.S., Mahmoud D.A., Abd El-Kareim M.S., Abdelgaleil S.A.M. (2021). Insecticidal activity of lemongrass (*Cymbopogon citratus*) essential oil as an eco-friendly agent against the black cutworm *Agrotis ipsilon* (Hufnagel). Insects.

[73] Carreño Otero A.L., Palacio-Cortés A.M., Navarro-Silva M.A., Kouznetsov V.V., Duque L.J.E. (2018). Behavior of detoxifying enzymes of *Aedes aegypti* (Diptera: Culicidae) exposed to girgensohnine alkaloid analog and *Cymbopogon flexuosus* essential oil. Comp. Biochem. Physiol. C Toxicol. Pharmacol..

[74] Tak J.H., Isman M.B. (2016). Metabolism of citral, the major constituent of lemongrass oil, in the cabbage looper, *Trichoplusia ni*, and effects of enzyme inhibitors on toxicity and metabolism. Pestic. Biochem. Physiol..

[75] Jankowska M., Rogalska J., Wyszkowska J., Stankiewicz M. (2018). Molecular targets for components of essential oils in the insect nervous system—A review. Molecules.

[76] Viteri Jumbo L.O., Moura W.S., Possel R.D., Herrera O.M., Fidelis R.R., Andrade B.S., Smagghe G., Santos G.R., Oliveira E.E., Aguiar R.W.S. (2025). Phytochemistry, mode of action predictions, and synergistic potential of *Hypenia irregularis* essential oil mixtures for controlling *Aedes aegypti*. Toxins.

[77] Qasim M., Islam W., Rizwan M., Hussain D., Noman A., Khan K.A., Ghramh H.A., Han X. (2024). Impact of plant monoterpenes on insect pest management and insect-associated microbes. Heliyon.

[78] Popescu I.E., Gostin I.N., Blidar C.F. (2024). An overview of the mechanisms of action and administration technologies of the essential oils used as green insecticides. AgriEngineering.

[79] Turchen L.M., Piton L.P., Oglio E.D., Butnariu A.R., Pereira M.J.B. (2016). Toxicity of *Piper aduncum* (Piperaceae) essential oil against *Euschistus heros* (F.) (Hemiptera: Pentatomidae) and non-effect on egg parasitoids. Neotrop. Entomol..

[80] Cossolin J.F.S., Pereira M.J.B., Martínez L.C., Turchen L.M., Fiaz M., Bozdogan H., Serrão J.E. (2019). Cytotoxicity of *Piper aduncum* (Piperaceae) essential oil in brown stink bug *Euschistus heros* (Heteroptera: Pentatomidae). Ecotoxicology.

[81] Chaudhari A.K., Singh V.K., Kedia A., Das S., Dubey N.K. (2021). Essential oils and their bioactive compounds as eco-friendly novel green pesticides for management of storage insect pests: Prospects and retrospects. Enviro. Sci. Pollut. Res..

[82] Santana M.L.G., Melo J.P.R., Camara C.A.G., Moraes M.M., Araujo C.A., Vasconcelos G.J.N., Pereira M.R.S., Zartman C.E. (2022). Lethal and sublethal effects of essential oils from *Piper capitarianum* Yunck and *Piper krukoffii* Yunck on *Plutella xylostella* L. An. Acad. Bras. Ciênc..

[83] Brugger B.P., Martínez L.C., Plata-Rueda A., Castro B.M.C., Soares M.A., Wilcken C.F., Carvalho A.G., Serrão J.E., Zanuncio J.C. (2019). Bioactivity of the *Cymbopogon citratus* (Poaceae) essential oil and its terpenoid constituents on the predatory bug, *Podisus nigrispinus* (Heteroptera: Pentatomidae). Sci. Rep..

[84] Nogueira J., Mourão S.C., Dolabela I.B., Santos M.G., Mello C.B., Kelecom A., Mexas R., Feder D., Fernandes C.P., Gonzalez M.S. (2014). *Zanthoxylum caribaeum* (Rutaceae) essential oil: Chemical investigation and biological effects on *Rhodnius prolixus* nymph. Parasitol. Res..

[85] Chauhan N., Malik A., Sharma S., Dhiman R.C. (2016). Larvicidal potential of essential oils against *Musca domestica* and *Anopheles stephensi*. Parasit. Res..

[86] Zanuncio J.C., Mourão S.A., Martínez L.C., Wilcken C.F., Ramalho F.S., Plata-Rueda A., Soares M.A., Serrão J.E. (2016). Toxic effects of the neem oil (*Azadirachta indica*) formulation on the stink bug predator, *Podisus nigrispinus* (Heteroptera: Pentatomidae). Sci. Rep..

[87] Pacheco J.P.F., Nogueira J., Miranda R.P.R., Duprat R.C., Machado F.P., Tietbohl L.A.C., Mourão S.C., Santos M.G., Ratcliffe N.A., Penna P.A. (2020). Effects of *Zanthoxylum caribaeum* essential oil against cotton bug *Dysdercus peruvianus*. Res. Soc. Dev..

[88] Taszakowski A., Kaszyca-Taszakowska N. (2020). Teratological cases of the antennae in the family Aradidae (Hemiptera: Heteroptera). Sci. Rep..

[89] Wang J.Z., Qi Y.T., Zhang J.W., Fei C., Yuan Y.K., Du S.S. (2023). Contact toxicity and repellent activity of essential oils from *Alpinia zerumbet* cv. ‘Variegata’ against stored product insects. Int. J. Food Prop..

[90] Gvozdenac S., Kiprovski B., Acimovic M., Jeremic J.S., Cvetkovic M., Bursic V., Ovuka J. (2021). Repellent activity of *Cymbopogon citratus* essential oil against four major stored product pests: *Plodia interpunctella*, *Sitophilus oryzae*, *Acanthoscelides obtectus* and *Tribolium castaneum*. Contemp. Agric..

[91] Devrnja N., Milutinovic M., Savic J. (2022). When scent becomes a weapon—Plant essential oils as potent bioinsecticides. Sustainability.

[92] Yang L., Yao X., Liu B., Han Y., Ji R., Ju J., Zhang X., Wu S., Fang J., Sun Y. (2022). Caterpillar-induced rice volatile (E)-β-Farnesene impairs the development and survival of *Chilo suppressalis* larvae by disrupting insect hormone balance. Front. Physiol..

[93] Corrêa E.J.A., Carvalho F.C., Oliveira J.A.C., Bertolucci S.K.V., Scotti M.T., Silveira C.H., Guedes F.C., Melo J.O.F., Melo-Minardi R.C., Lima L.H.F. (2023). Elucidating the molecular mechanisms of essential oils’ insecticidal action using a novel cheminformatics protocol. Sci. Rep..

[94] Ebihara K., Niwa R. (2023). Compounds inhibiting Noppera-bo, a glutathione S-transferase involved in insect ecdysteroid biosynthesis: Novel Insect Growth Regulators. Biomolecules.

[95] Henriques B.S., Genta F.A., Mello C.B., Silva L.R., Codogno T.F., Oliveira A.F.R., Marinho L.P., Valle D., Lima J.B.P., Feder D. (2016). Triflumuron effects on the physiology and reproduction of *Rhodnius prolixus* adult females. BioMed Res. Int..

[96] FAO, World Health Organization (2020). Pesticide Residues in Food 2019—Report 2019—Joint FAO/WHO Meeting on Pesticide Residues.

[97] Ganguly P., Mandal J., Mandal N., Rakshit R., Patra S. (2020). Benzophenyl urea insecticides—Useful and eco-friendly options for insect pest control. J. Environ. Biol..

[98] Bajerski L., Michels L.R., Colomé L.M., Bender E.A., Freddo R.J., Bruxel F., Haas S.E. (2016). The use of Brazilian vegetable oils in nanoemulsions: An update on preparation and biological applications. Braz. J. Pharm. Sci..

[99] FAO (2018). FAO Specifications and Evaluations for Agricultural Pesticides—Triflumuron.

[100] Martínez L.C., Plata-Rueda A., Serrão J.E. (2021). Effect of benzoylphenyl ureas on survival and reproduction of the lace bug, *Leptopharsa gibbicarina*. Insects.

[101] Koczur W., Szwedo J., Golebiowski M. (2024). The defensive secretion of *Eurycantha calcarata* (Phasmida: Lonchodidae)—Chemical composition and method of collection. Eur. J. Entomol..

[102] Vaníčkova L., Nascimento R.R., Hoskovec M., Jezková Z., Brizová R., Tomcala A., Kalinová B. (2012). Are the wild and laboratory insect populations different in semiochemical emission? The case of the medfly sex pheromone. J. Agric. Food Chem..

[103] Mendki J., Ganesan K., Parashar B.D., Sukumaran D., Prakash S. (2014). Aggregation responses of *Cimex hemipterus* F. to semiochemicals identified from their excreta. J. Vector Borne Dis..

[104] Ryu C.M., Farag M.A., Hu C.H., Reddy M.S., Wei H.X., Paré P.W., Kloepper J.W. (2003). Bacterial volatiles promote growth in *Arabidopsis*. Proc. Natl. Acad. Sci. USA.

[105] Saïd I., Renou M., Morin J.P., Ferreira J.M.S., Rochat D. (2005). Interactions between acetoin, a plant volatile, and pheromone in *Rhynchophorus palmarum*: Behavioral and olfactory neuron responses. J. Chem. Ecol..

[106] Engl T., Kaltenpoth M. (2018). Influence of microbial symbionts on insect pheromones. Nat. Prod. Rep..

[107] Aldrich J.R. (1988). Chemical ecology of the Heteroptera. Annu. Rev. Entomol..

[108] Picciotti U., Valverde-Urrea M., Garganese F., Lopez-Moya F., Foubelo F., Porcelli F., Lopez-Llorca L.V. (2023). Brindley’s glands volatilome of the predator *Zelus renardii* interacting with *Xylella* vectors. Insects.

[109] Akhoundi M., Chebbah D., Elissa N., Brun S., Jan J., Lacaze I., Izri A. (2023). Volatile organic compounds: A promising tool for bed bug detection. Int. J. Environ. Res. Public Health.

[110] Xu T., Hansen L., Cha D., Hao D., Zhang L., Teale S.A. (2022). Identification of a female-produced pheromone in a destructive invasive species: Asian longhorn beetle, *Anoplophora glabripennis*. J. Pest. Sci..

[111] Ashbrook A.R., Feder J.L., Bennett G.W., Ginzel M.D., Gondhalekar A.D. (2024). Lethal and sublethal heat-exposure of bed bugs (*Cimex lectularius* L.) causes alarm pheromone emission and elicits a movement response in nearby recipients. Sci. Rep..

[112] Berville L., Lucas C., Haouzi M., Khalil A., Gévar J., Bagnères A.G., Darrouzet E. (2023). Chemical profiles of venom glands in queens, foundresses, pre-wintering gynes, and workers in the hornet *Vespa velutina nigrithorax*. C. R. Chim..

[113] Lacey E.S., Moreira J.A., Millar J.G., Hanks L.M. (2008). A male-produced aggregation pheromone blend consisting of alkanediols, terpenoids, and an aromatic alcohol from the cerambycid beetle *Megacyllene caryae*. J. Chem. Ecol..

[114] Kempraj V., Park S.J., Cameron D.N.S., Taylor P.W. (2022). 1-Octanol emitted by *Oecophylla smaragdina* weaver ants repels and deters oviposition in Queensland fruit fly. Sci. Rep..

[115] Meier L.R., Zou Y., Mongold-Diers J.A., Millar J.G., Hanks L.M. (2020). Pheromone composition and chemical ecology of six species of cerambycid beetles in the subfamily Lamiinae. J. Chem. Ecol..

[116] Kerr J.L., Romo C.M., O’Connor B., Dickson G., Novoselov M., Aguilar-Aguello S., Todoroki C., Najar-Rodriguez A., Manning L.A., Twidle A. (2024). Exploring the nature of *Arhopalus ferus* (Coleoptera: Cerambycidae: Spondylidinae) pheromone attraction. J. Chem. Ecol..

[117] Tholl D., Rebholz Z., Morozov A.V., O’Maille P.E. (2023). Terpene synthases and pathways in animals: Enzymology and structural evolution in the biosynthesis of volatile infochemicals. Nat. Prod. Rep..

[118] Jayaram C.S., Chauhan N., Dolma S.K., Reddy S.G.E. (2020). Deformation of appendages, antennal segments and sensilla of aphid (*Aphis craccivora* Koch) treated with *Tagetes minuta* oil: A scanning electron microscopy study. Toxin Rev..

[119] Zhang Y., Zhang T., Wang X., Bian Z., Zhang X., Yang G., Lu Y. (2024). Volatiles from essential oils of three Lamiaceae plants repel the winged cotton aphid, disturb its feeding behavior and reduce its fecundity. Pest Manag. Sci..

