# Supplementary material for: Insecticidal and Ovicidal Activity of Cymbopogon citratus Essential Oil and Its Nanoemulsion Against Hemipteran Crop Pests with Mortality, Antennal Malformations, and Volatile Alterations
Source: Insects. 2025 Dec 10;16(12):1254. doi: 10.3390/insects16121254 (PMC12734222; doi:10.3390/insects16121254)
Supplement: Supplementary file 1 [file insects-16-01254-s001.zip › insects-3978012-supplementary.pdf]

Insecticidal and Ovicidal Activity of *Cymbopogon citratus* Essential Oil and Its Nanoemulsion Against Hemipteran Crop Pests with Mortality, Antennal Malformations, and Volatile Alterations

Raul V. C. Apolinário [1], Jefferson D. Cruz [1], Walter S. M. F. Neto [2], Janaína M. C. Soares [2], Maria A. Mpalantinos [1], Suzete A. Gomes [3], Maria D. Feder [3], José L. P. Ferreira [4], Geraldo J. N. Vasconcelos [5], Jefferson R. A. Silva [2,*] and Ana Claudia F. Amaral [1,*]

1 Laboratório de Plantas Medicinais e Derivados, Farmanguinhos, Fundação Oswaldo Cruz, Rio de Janeiro 21041-250, RJ, Brazil; apolinario_raul@yahoo.com (R.V.C.A.); jefferson_dacruz@hotmail.com (J.D.C.); maria.mpalantinos@fiocruz.br (M.A.M.)

2 Laboratório de Cromatografia, Departamento de Química, Instituto de Ciências Exatas, Universidade Federal do Amazonas, Manaus 69077-000, AM, Brazil; wssottoo@gmail.com (W.S.M.F.N.); janaina25soares02@gmail.com (J.M.C.S.)

3 Departamento de Biologia Geral—GBG, Instituto de Biologia, Universidade Federal Fluminense, Niterói 24210-201, RJ, Brazil; suzetearaujo@id.uff.br (S.A.G.); mdfeder@id.uff.br (M.D.F.)

4 Departamento de Farmácia e Administração Farmacêutica, Faculdade de Farmácia, Universidade Federal Fluminense, Niterói 24241-000, RJ, Brazil; josepint06@yahoo.com.br

5 Instituto de Ciências Exatas e Tecnologia, Universidade Federal do Amazonas, Itacoatiara 69103-128, AM, Brazil; gjnvasconcelos@ufam.edu.br

* Correspondence: jrocha_01@yahoo.com.br (J.R.A.S.); aamaral_99@yahoo.com.br (A.C.F.A.)

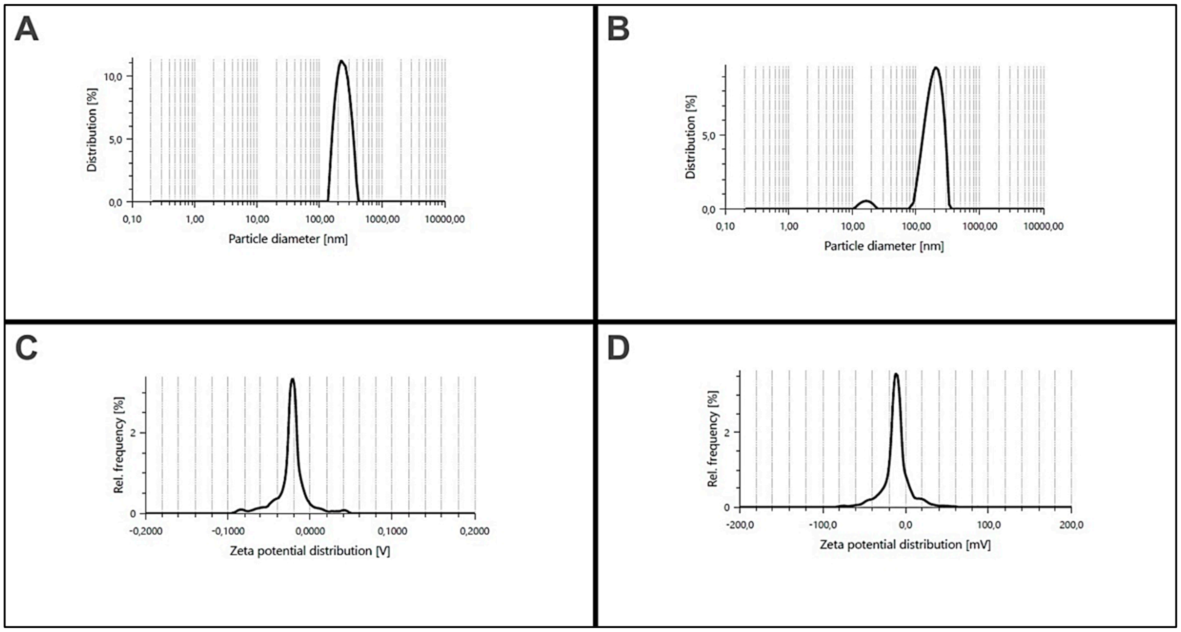

Figure S1. HLB 14 nanoemulsion characterization. Particle size distribution: (A) *Cymbopogon citratus* essential oil nanoemulsion (242 nm) and (B) blank nanoemulsion without essential oil (195.11 nm). Zeta potential distribution: (C) essential oil nanoemulsion (-22.1 mV) and (D) blank nanoemulsion (-9.6 mV)

Table S1. Percentage of deformations observed in *Dysdercus peruvianus* and *Euschistus heros* insects after topical and contact treatments with *Cymbopogon citratus* essential oil, oil nanoemulsion, blank nanoemulsion (without essential oil), positive control (triflumuron) and their respective untreated controls. Percentages were calculated based on the total number of insects used in each treatment group (n = 90 insects). Values expressed as % ± SD

	Dysdercus peruvianus						*Euschistus heros*					
	**Topical treatment**			**Contact treatment**			**Topical treatment**			**Contact treatment**		
**Experimental groups**	**AM**	**FI-Ex**	**A-Ex**	**AM**	**FI-Ex**	**A-Ex**	**AM**	**FI-Ex**	**A-Ex**	**AM**	**FI-Ex**	**A-Ex**
Pure **EO**	0	0	0	0	0	0	0	0	0	0	0	0
**EO** nanoemulsion	0	0	0	64.44 ± 2.18*	4.44 ± 0.53	0	0	0	4.44 ± 0.73	36.67 ± 2.40*	0	0
Untreated control	0	0	0	0	0	0	0	0	0	0	0	0
Blank nanoemulsion	0	0	0	0	0	0	0	0	0	0	0	0
Positive control	0	45.56 ± 2.65*	3.33 ± 0.5	0	3.33 ± 0.71	1.11 ± 0.33	0	17.10 ± 1.61*	24.30 ± 1.73*	0	0	2.22 ± 0.44
**ANOVA**	-	F(4,40) = 26.58	F(4,40) = 4.00	F(4,40) = 78.23	F(4,40) = 2.71	F(4,40) = 1.00	-	F(4,40) = 15.36	F(4,40) = 21.76	F(4,40) = 21.04	-	F(4,40) = 2.29

EO – essential oil. **AM** = Antennae malformation. **FI-Ex** = Fifth-instar in exuvia. **A-Ex** = Adult in exuvia. * Statistical relevant data related to comparison with untreated control = P <0.0001.

Table S2. Concentrations of volatile compounds (ppb) detected in *Euschistus heros* and *Dysdercus peruvianus* by HS-SPME/GC-MS analysis. Data from untreated control samples and samples treated with *Cymbopogon citratus* essential oil nanoemulsion.

**Compounds**$_{(CAS)}$	**RI**	***Euschistus heros***		***Dysdercus peruvianus***	
		**Untreated**	**Treated**	**Untreated**	**Treated**
**Alcohols**					
3-Methyl-1-butanol$_{123-51-3}$	726	-	8706.8	-	-
2,3-Butanediol$_{513-85-9}$	782	-	63.6	-	-
1-Hexanol$_{111-27-3}$	863	-	-	109.5	-
3-Methylthiopropanol$_{505-10-2}$	977	-	157.1	-	-
6-Methylhept-5-en-2-ol$_{1569-60-4}$	993	-	445.3	-	866.6
2-Ethyl-1-hexanol$_{104-76-7}$	1029	-	579.0	-	-
2-Octen-1-ol$_{22104-78-5}$	1040	539.9	-	138.9	-
3-Octen-1-ol$_{18185-81-4}$	1044	-	-	148.5	283.2
Phenylethyl alcohol$_{60-12-8}$	1060	-	-	194.4	-
1-Octanol$_{111-87-5}$	1063	-	-	70.3	-
p-Menth-1-en-8-ol$_{98-55-5}$	1199	-	63.0	-	-
Dodecanol$_{112-53-8}$	1469	-	-	37.1	-
2,4-Dimethylhexan-3-ol$_{13432-25-2}$	-	-	360.3	-	-
**Aldehydes**					
2-Methylbutanal$_{96-17-3}$	661	-	22077.9	-	-
Benzaldehyde$_{100-52-7}$	964	-	185.8	-	-
Octanal$_{124-13-0}$	1000	-	-	78.9	-
Benzeneacetaldehyde$_{122-78-1}$	1041	-	854.8	246.9	-
(E)-2-octenal$_{2548-87-0}$	1058	3979.2	-	815.4	-
Nonanal$_{124-19-6}$	1102	-	-	131.9	759.3
Decanal$_{112-31-2}$	1214	-	45.9	68.8	-
(Z)-2-Decenal$_{2497-25-8}$	1250	312.5	-	-	-
**Ketones**					

Acetoin$_{513-86-0}$	706	14102.3	-	-	-
3-Methylhexan-2-one$_{2550-21-2}$	832	-	-	-	3447.0
4-Methyl-2-hexanone$_{105-42-0}$	850	-	125.8	-	-
2-Heptanone$_{110-43-0}$	900	-	105.5	-	-
2,2-Dimethyl-3-heptanone$_{19078-97-8}$	963	-	451.2	-	-
6-Methyl-5-hepten-2-one$_{110-93-0}$	989	-	-	-	541.5
3-Methyl-2-cyclohexen-1-one$_{1193-18-6}$	1026	-	-	-	641.9
4-Methyl-6-hepten-3-one$_{26118-97-8}$	1113	-	295.6	-	-
2,11-Dodecanedione$_{7029-09-6}$	-	-	-	-	666.6
2,9-Decanedione$_{16538-91-3}$	-	-	-	1473.7	39.8
**Organic acids**					
2-Methylbutanoic acid$_{116-53-0}$	876	-	1659.6	-	-
3-Hexenoic acid$_{4219-24-3}$	1023	1007.7	-	-	-
Hexanoic acid$_{142-62-1}$	1026	152.4	-	176.1	-
2-Ethylhexanoic acid$_{149-57-5}$	1123	-	-	14.0	-
2-Octenoic acid$_{1871-67-6}$	1245	1697.6	-	-	-
Geranic acid$_{459-80-3}$	1355	-	-	-	1057.1
**Ester**					
Ethyl isobutyrate$_{97-62-1}$	751	-	1166.4	-	-
Ethyl butyrate$_{105-54-4}$	799	-	174.9	-	-
Ethyl isovalerate$_{108-64-5}$	856	-	4959.3	-	-
Ethyl 2-hexenoate$_{1552-67-6}$	1038	-	189.7	-	-
Ethyl 2-ethylhexanoate$_{2983-37-1}$	1098	827.4	-	-	-
1-Octen-3-yl acetate$_{2442-10-6}$	1110	120.4	-	-	-
Octanoic acid, methyl ester$_{111-11-5}$	1129	224.0	-	-	-
Methyl oct-2-enoate$_{2396-85-2}$	1164	1034.3	-	-	-
Ethyl octanoate$_{106-32-1}$	1190	87.3	81.1	-	-
Ethyl 4-methyloctanoate $_{56196-53-3}$	1267	469.2	502.1	-	-
Methyl 2-hexenoate$_{2396-77-2}$	1272	1473.1	-	-	-

Hexadecanoic acid, methyl ester112-39-0	1927	-	-	16.3	441.4
Methyl 3,3-dimethylbutyrate 10250-48-3	-	1166.2	-	-	-
**Terpenes**					
β-Pinene127-91-3	974	322.0	-	-	-
a-Phellandrene99-83-2	1002	168.1	-	-	-
p-Cymene99-87-6	1020	1359.4	-	-	640.3
Limonene138-86-3	1024	260.3	-	170.7	573.2
γ-Terpinene99-85-4	1054	-	-	-	836.5
Linalool oxide cis5989-33-3	1067	-	253.8	-	503.3
Linalool oxide trans34995-77-2	1084	-	-	-	426.0
Linalool78-70-6	1095	-	2793.1	-	-
Thymol89-83-8	1289	-	-	-	393.3
α-Copaene3856-25-5	1374	-	-	50.9	942.0
β-Elemene515-13-9	1389	-	-	-	150.2
α-Gurjunene489-40-7	1409	-	-	-	433.2
trans-Caryophyllene87-44-5	1417	1046.6	102.8	1153.5	3447.7
a-Guaiene3691-12-1	1437	-	-	-	226.2
α-Humulene6753-98-6	1452	-	-	400.2	205.8
β-Selinene17066-67-0	1489	-	-	-	584.4
δ-Cadinene483-76-1	1522	-	-	22.3	-
Caryophyllene oxide1139-30-6	1582	-	-	36.7	-
**Alkanes**					
Undecane1120-21-4	1100	1029.4	-	-	-
Dodecane112-40-3	1200	3534.7	248.1	-	-
1-Tridecene2437-56-1	1287	977.3	-	-	-
Tridecane629-50-5	1300	19592.7	15110.7	234.8	276.2
Tetradecane629-59-4	1400	1671.6	-	26.9	-
10,10-Dimethyl-2,6-dimethylenebicyclo[7.2.0]undecane 357414-37-0	1440	-	-	27.8	-

Pentadecane$_{629-62-9}$	1500	1368.4	574.8	-	-
Octadecane$_{593-45-3}$	1800	-	-	48.8	-
Nonadecane$_{629-92-5}$	1900	-	-	-	199.1
**Other compounds**					
2,5-Dimethylpyrazine$_{123-32-0}$	910	-	191.6	-	-
Phenol$_{108-95-2}$	992	185.2	-	-	634.6
2,4,6-Trimethylpyridine$_{108-75-8}$	996	-	212.5	-	-
5-Ethyl-3-hydroxy-4-methyl-2(5H)-furanone$_{698-10-2}$	1196	617.4	-	-	-
γ-Hexalactone$_{695-06-7}$	1036	199.8	-	-	-
Tetrahydro-6-propyl-2H-pyran-2-one$_{698-76-0}$	1288	-	-	520.6	-
g-Nonalactone$_{104-61-0}$	1363	-	-	11.0	-
Dillapiole$_{484-31-1}$	1620	223.8	-	-	-

RI: Retention Index
